# Supplementary figures and images for: Data-driven detection of age-related arbitrary monotonic changes in single-cell gene expression distributions
Source: PeerJ. 2024 Feb 8;12:e16851. doi: 10.7717/peerj.16851 (PMC10859082; doi:10.7717/peerj.16851)

correlation coefficient

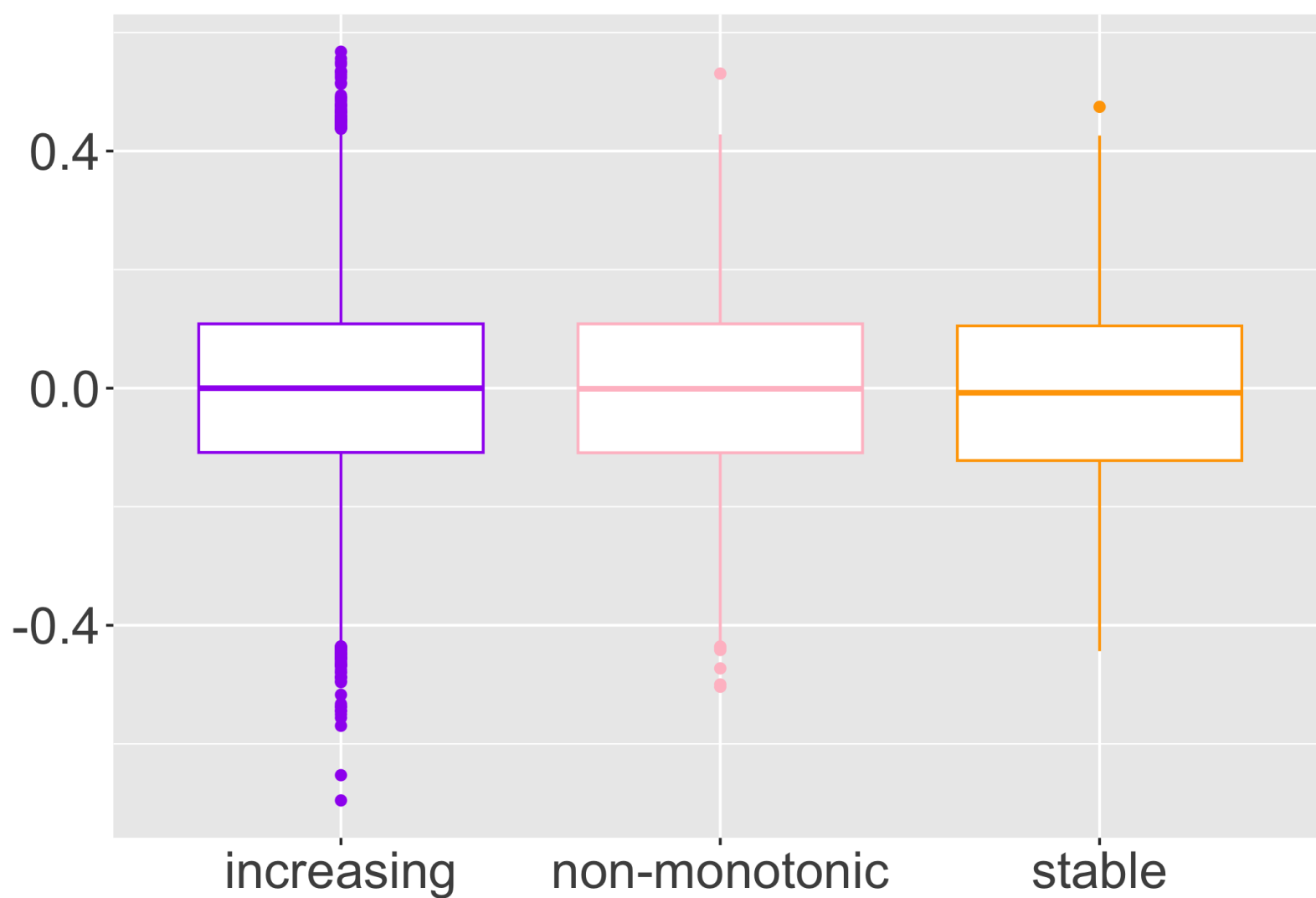

group

- increasing
- non-monotonic
- stable

Supplement: Supplemental Information 1 [file peerj-12-16851-s001.pdf]

(A)

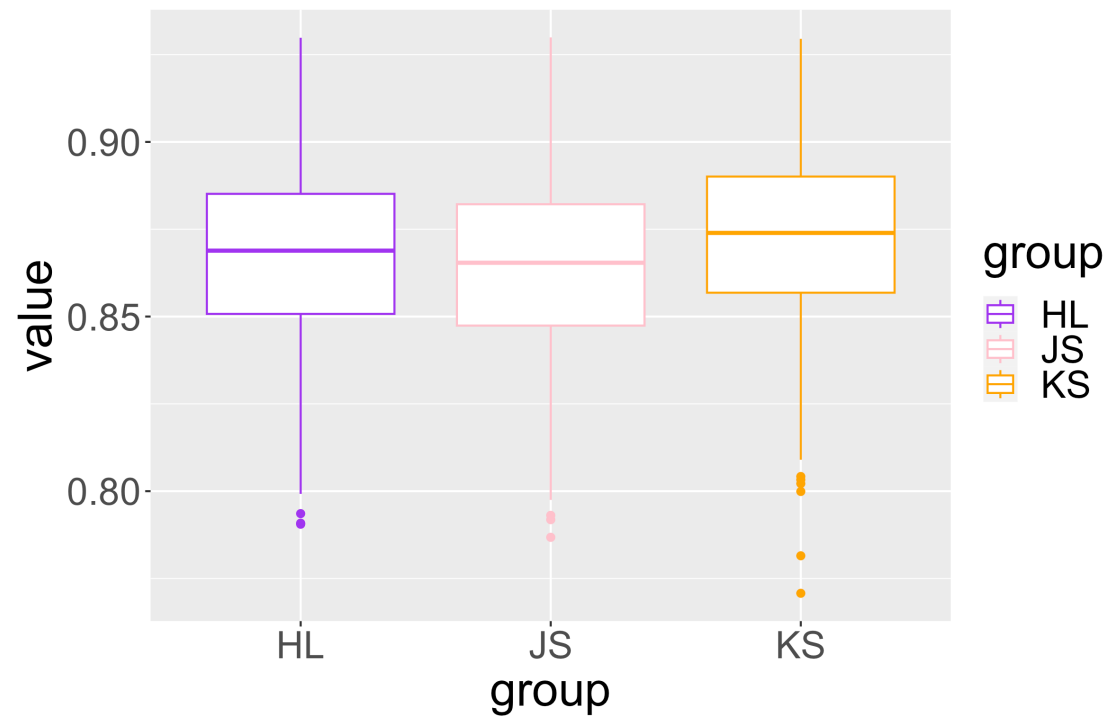

(B)

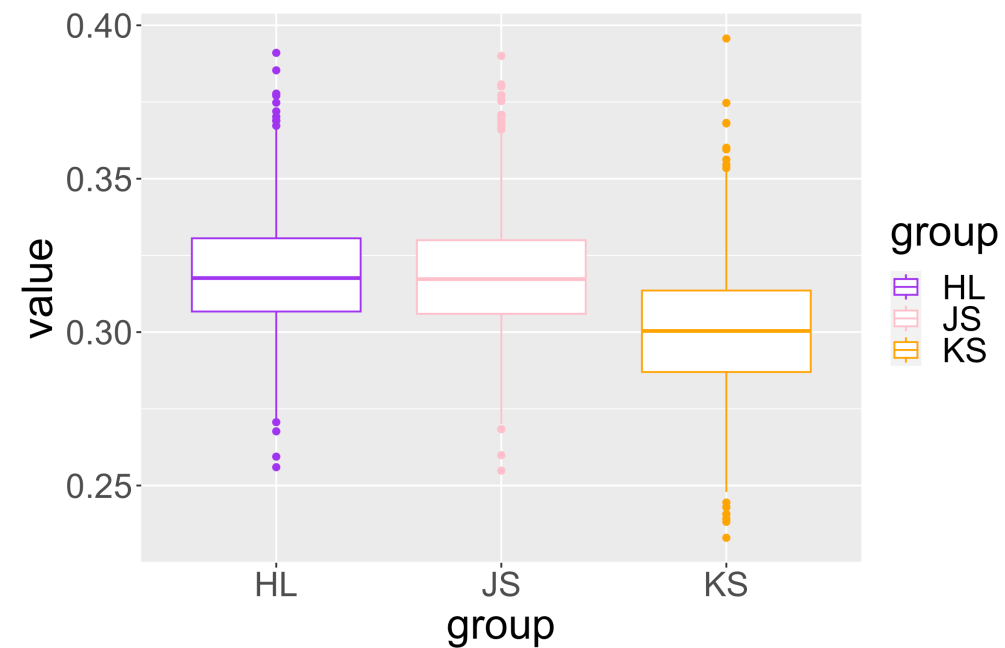

(C)

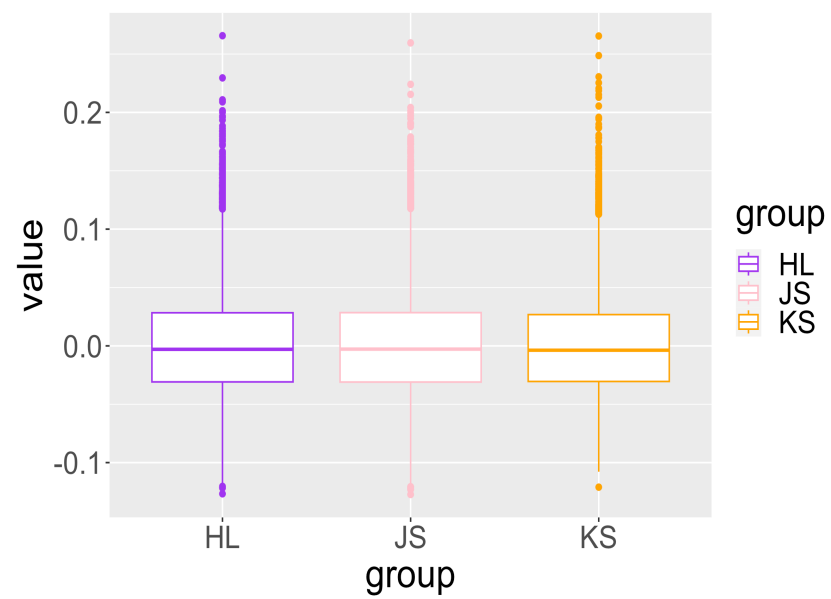

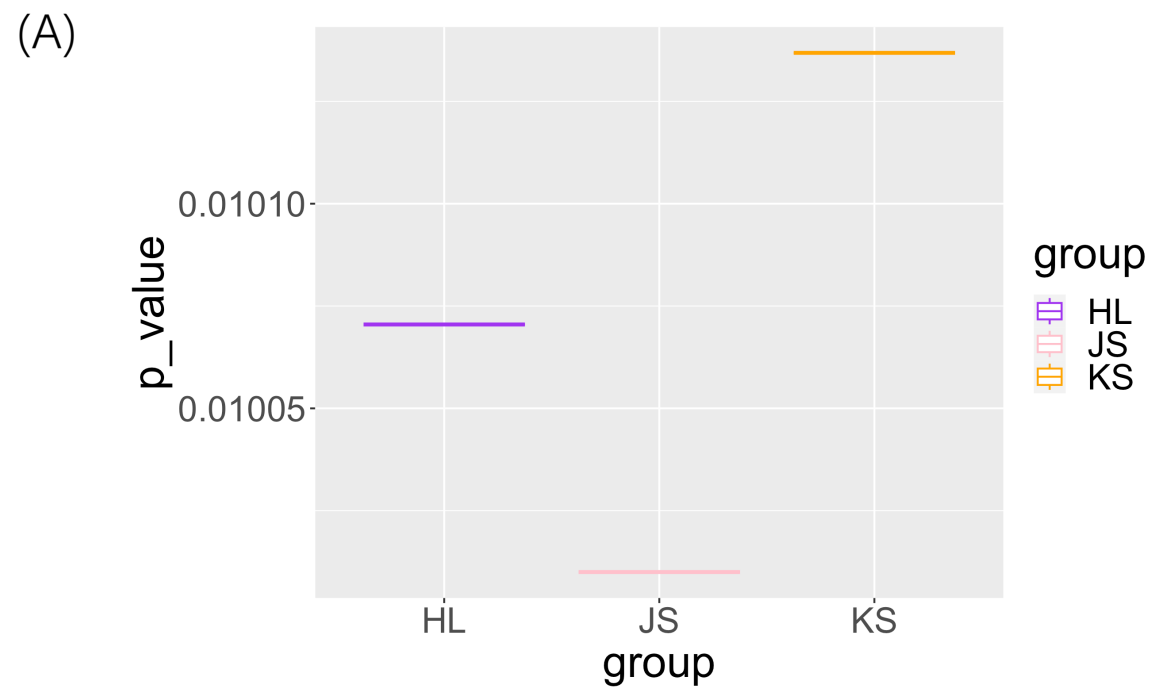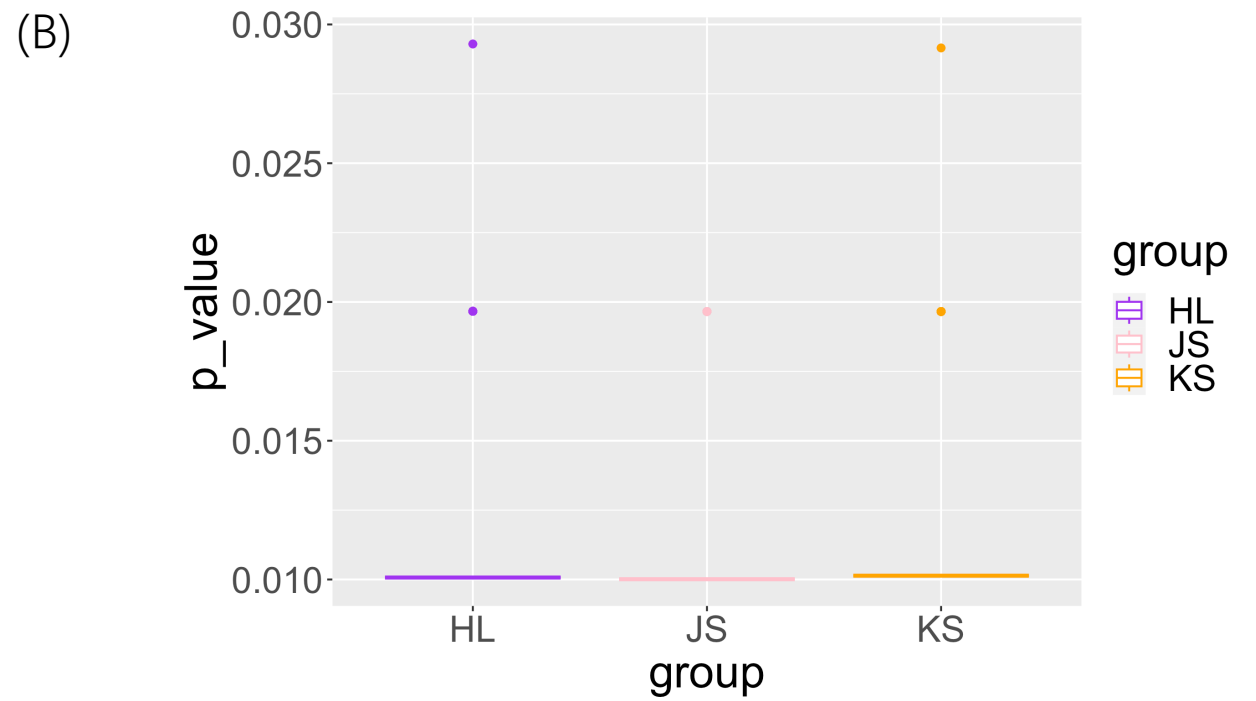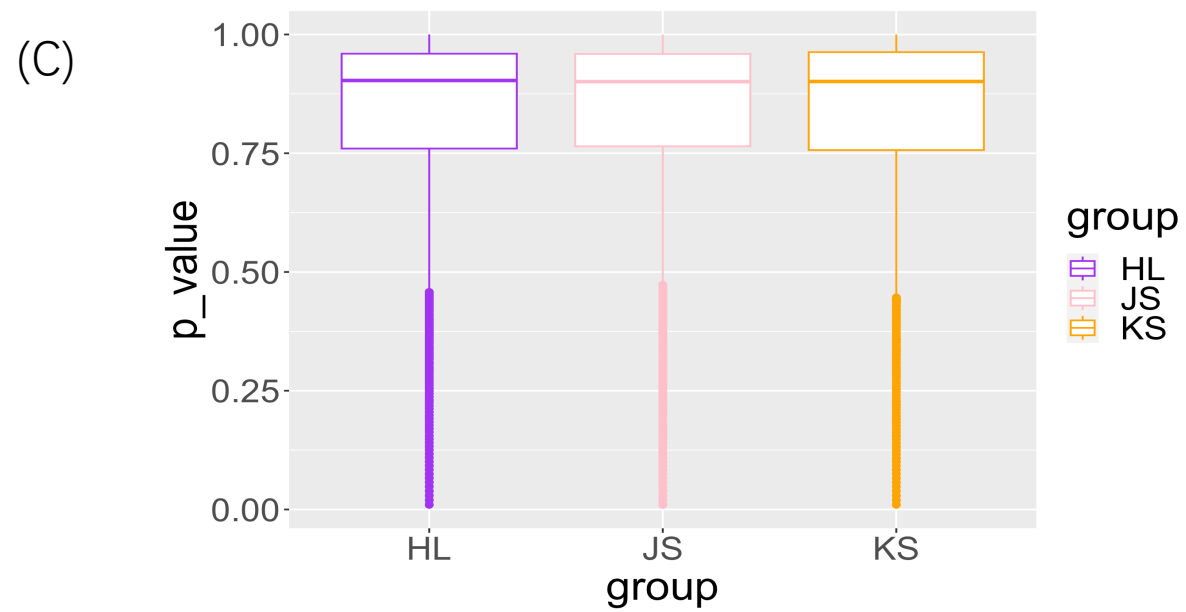

Supplement: Supplemental Information 2 — A box-and-whisker plot of the Spearman correlation coefficient and P-value for each aging pattern is described. [file peerj-12-16851-s002.pdf]

(A)

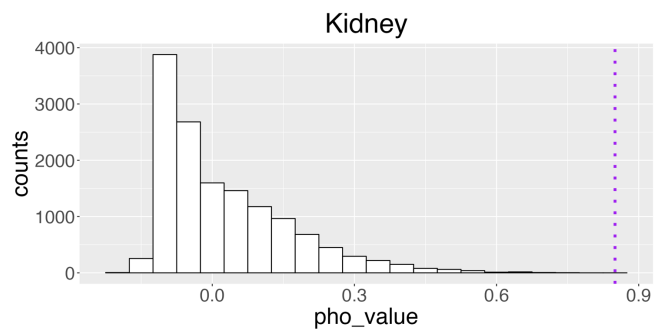

(B)

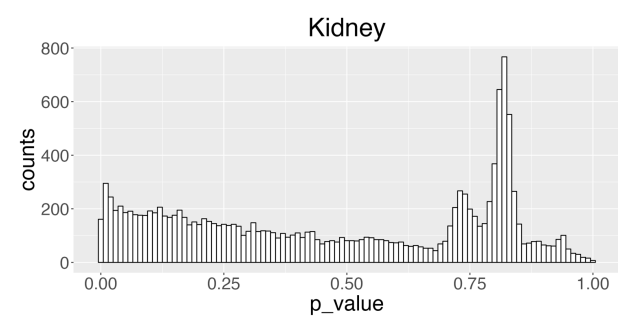

(C)

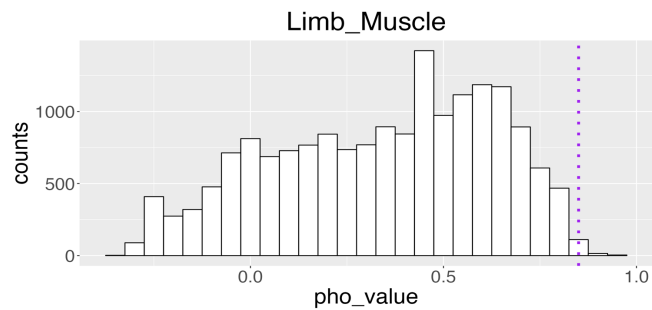

(D)

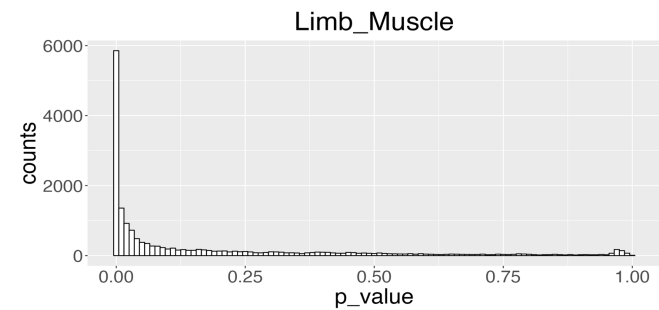

(E)

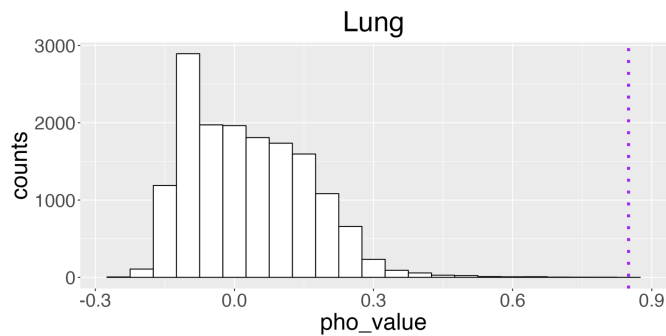

(F)

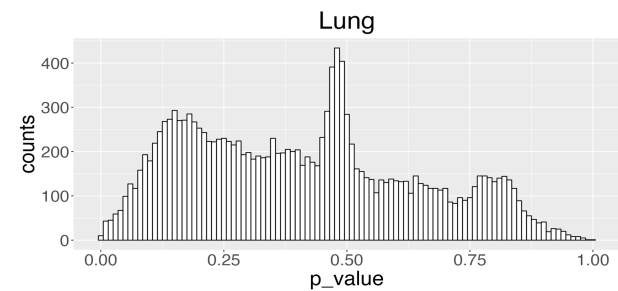

(G)

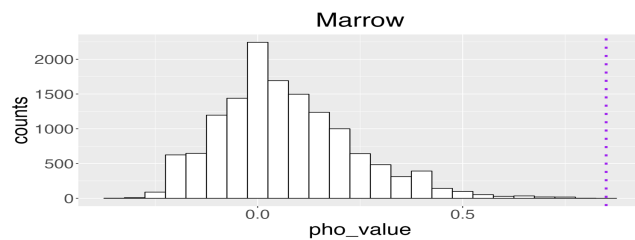

(H)

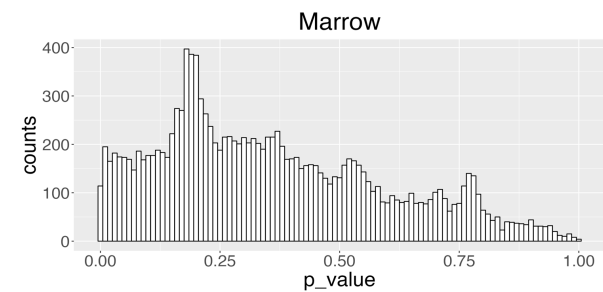

Supplement: Supplemental Information 5 [file peerj-12-16851-s005.pdf]
