# Supplementary material for: Data-driven detection of age-related arbitrary monotonic changes in single-cell gene expression distributions
Source: PeerJ. 2024 Feb 8;12:e16851. doi: 10.7717/peerj.16851 (PMC10859082; doi:10.7717/peerj.16851)
Supplement: Supplemental Information 3 — The x-axis represents the distribution at the youngest age and the y-axis represents the distribution at other ages. Cells from all samples in each age category are pooled. [file peerj-12-16851-s003.pdf]

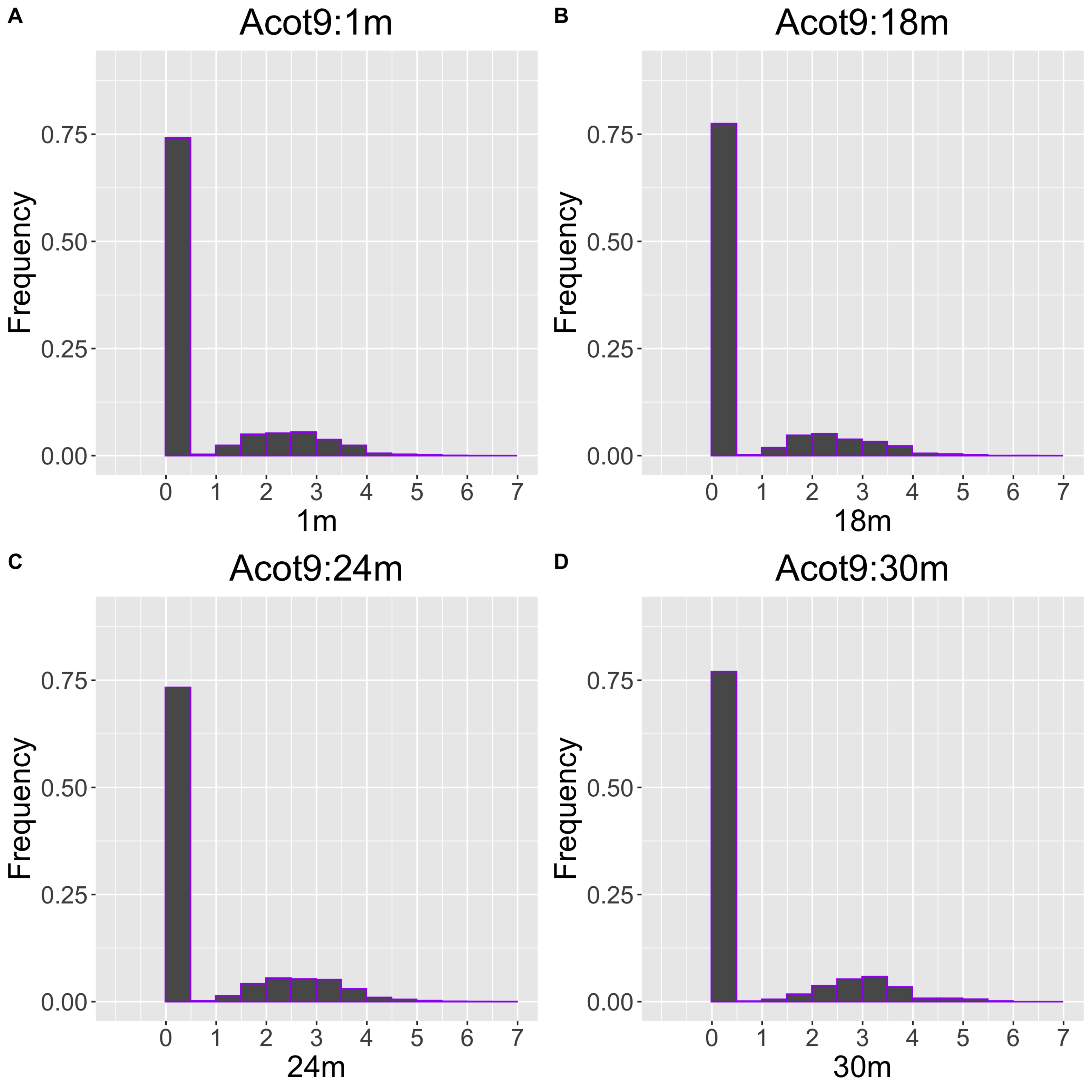

A
Acot9:1m
B
Acot9:18m
C
Acot9:24m
D
Acot9:30m
Frequency
0.00
0.25
0.50
0.75
0 1 2 3 4 5 6 7
1m
18m
24m
30m

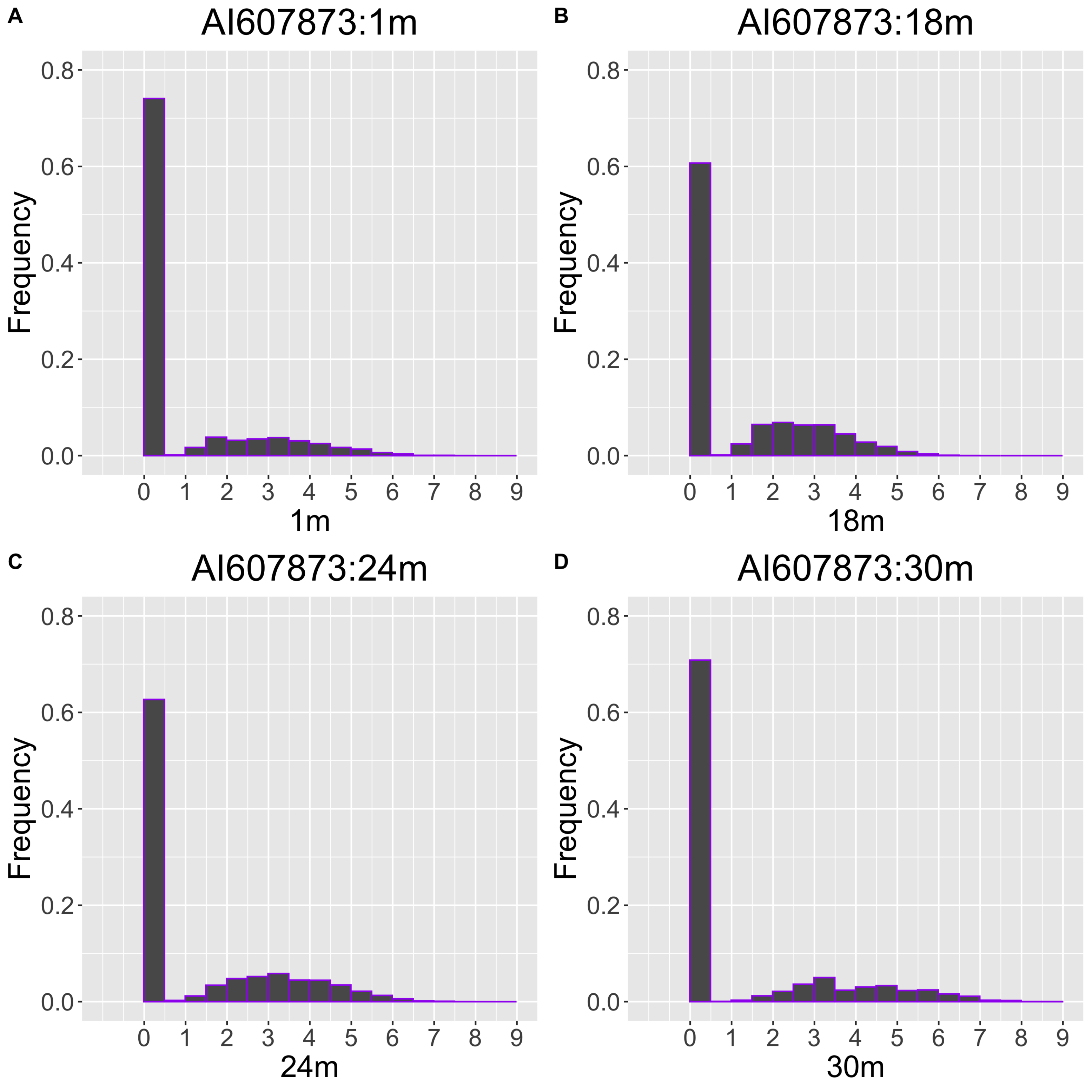
A
AI607873:1m
B
AI607873:18m
C
AI607873:24m
D
AI607873:30m
Frequency
0.0
0.2
0.4
0.6
0.8
0 1 2 3 4 5 6 7 8 9
1m
18m
24m
30m

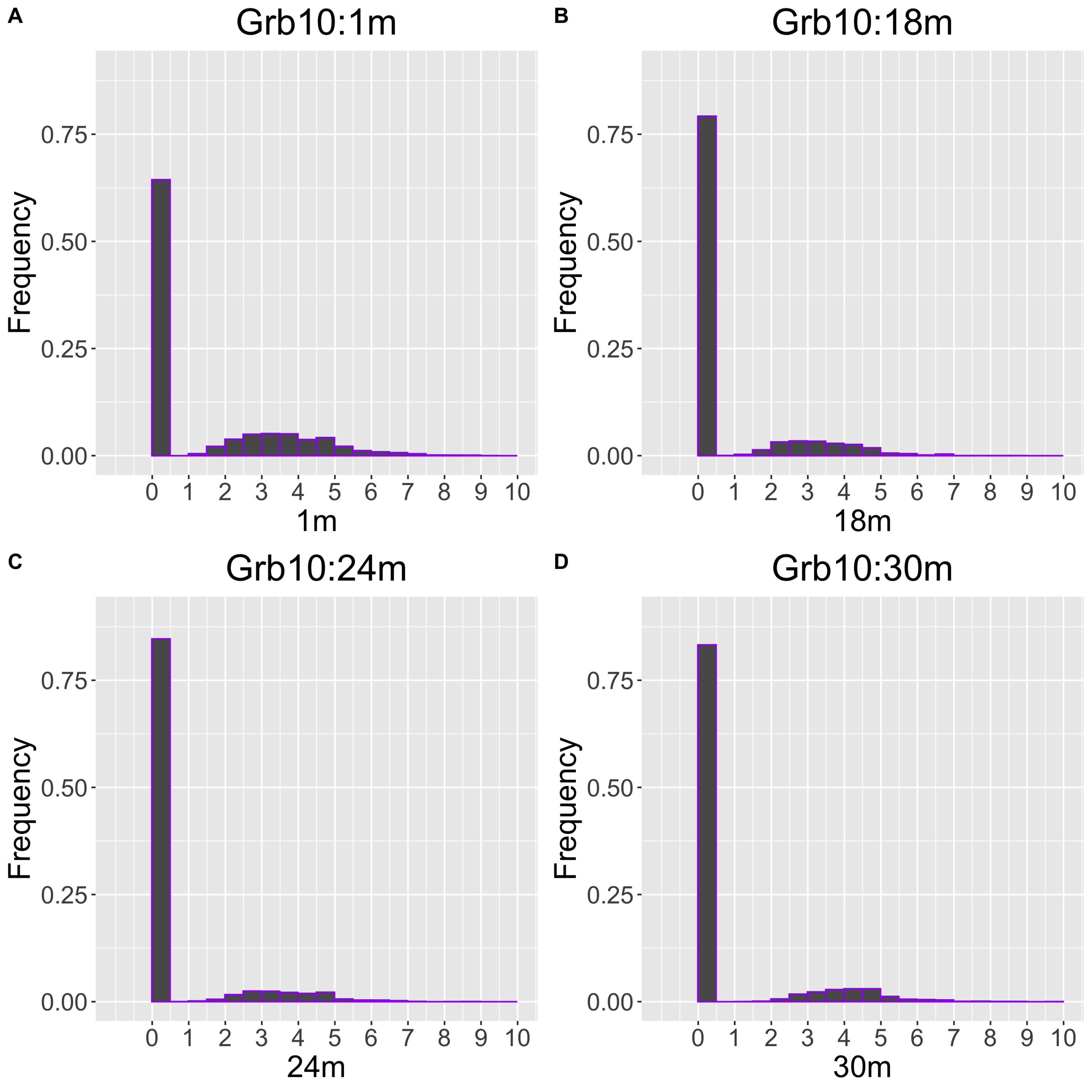
A
Grb10:1m
Frequency
0.00
0.25
0.50
0.75
0 1 2 3 4 5 6 7 8 9 10
1m
B
Grb10:18m
Frequency
0.00
0.25
0.50
0.75
0 1 2 3 4 5 6 7 8 9 10
18m
C
Grb10:24m
Frequency
0.00
0.25
0.50
0.75
0 1 2 3 4 5 6 7 8 9 10
24m
D
Grb10:30m
Frequency
0.00
0.25
0.50
0.75
0 1 2 3 4 5 6 7 8 9 10
30m

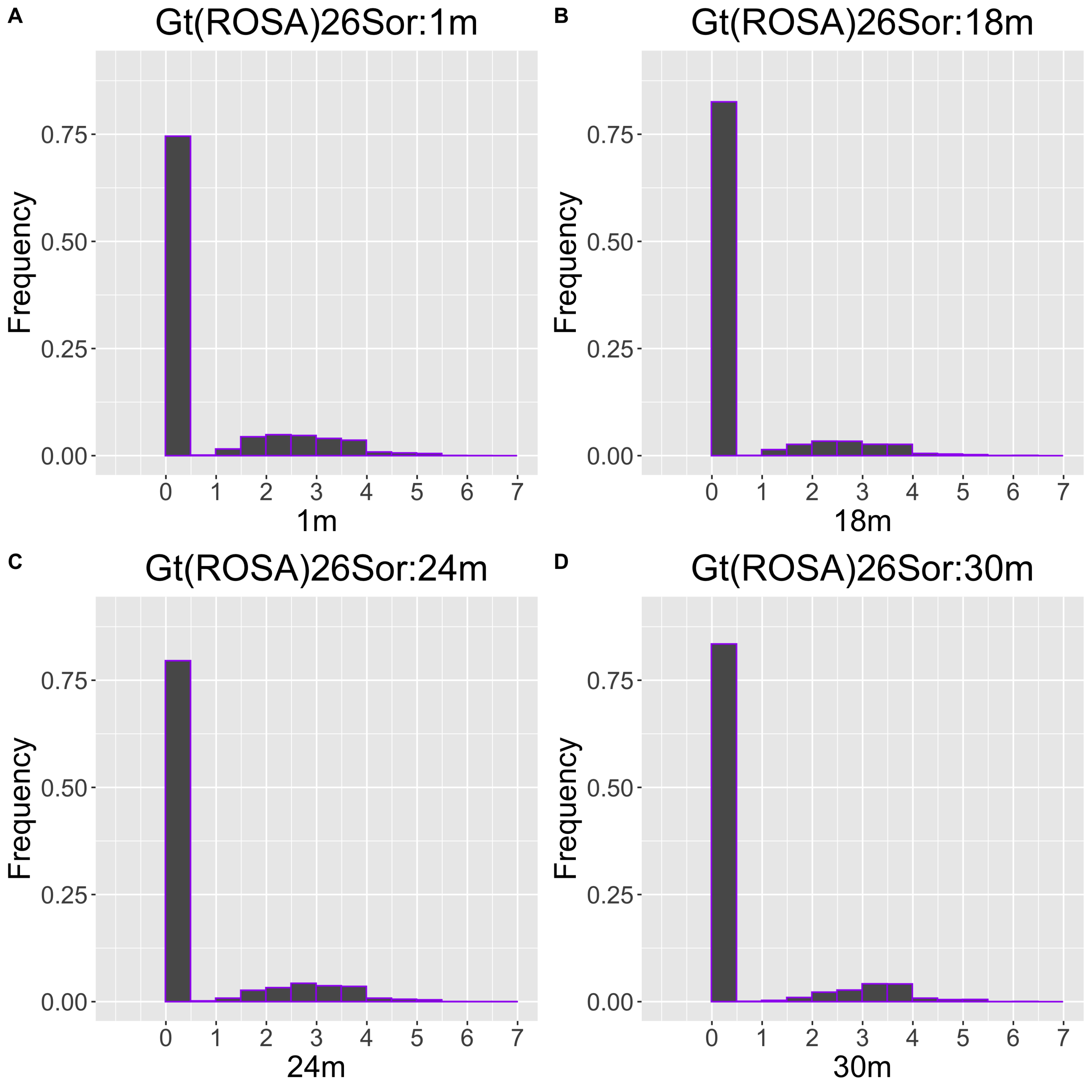
A
Gt(ROSA)26Sor:1m
Frequency
0.00
0.25
0.50
0.75
0
1
2
3
4
5
6
7
1m
B
Gt(ROSA)26Sor:18m
Frequency
0.00
0.25
0.50
0.75
0
1
2
3
4
5
6
7
18m
C
Gt(ROSA)26Sor:24m
Frequency
0.00
0.25
0.50
0.75
0
1
2
3
4
5
6
7
24m
D
Gt(ROSA)26Sor:30m
Frequency
0.00
0.25
0.50
0.75
0
1
2
3
4
5
6
7
30m

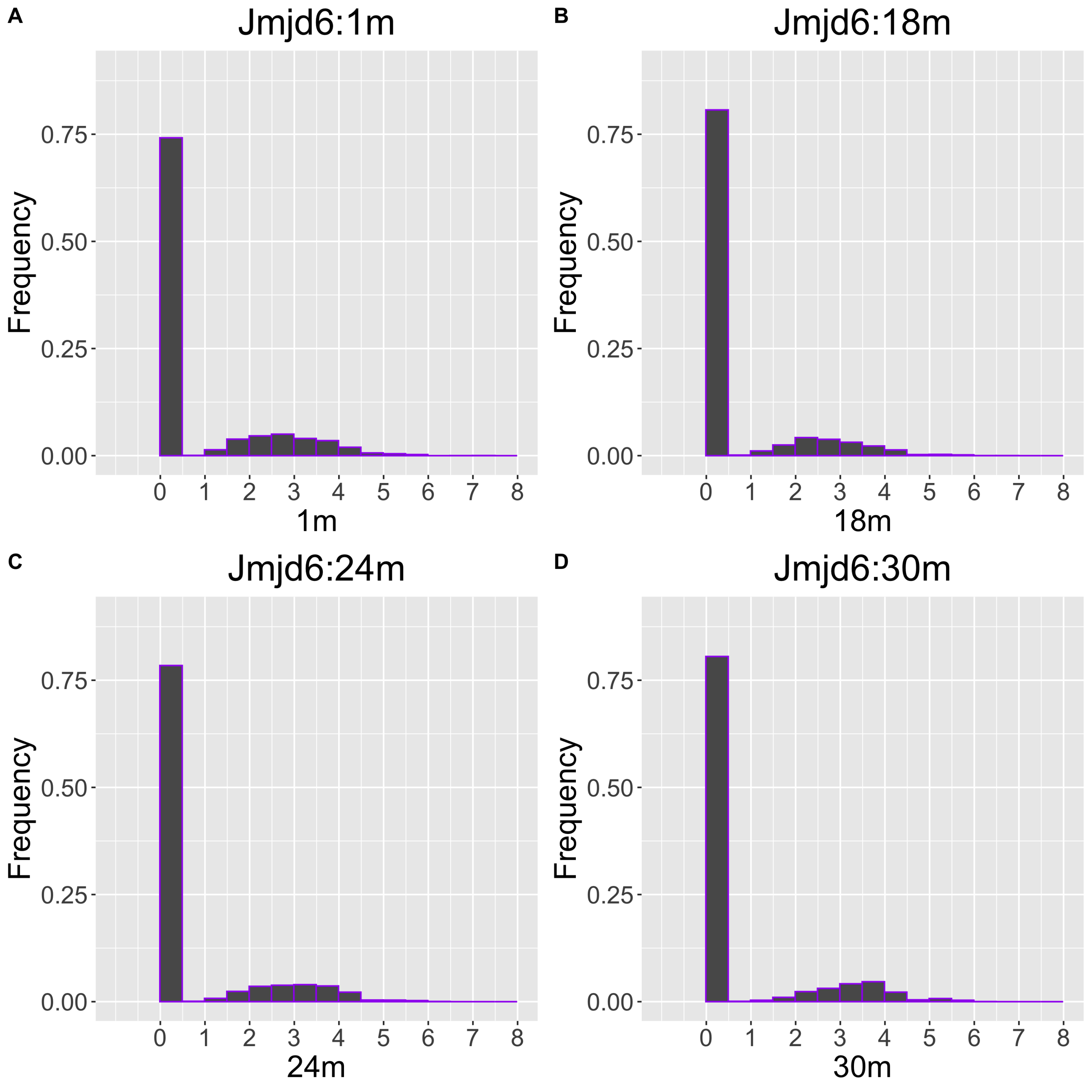

A
Jmjd6:1m
Frequency
0.75
0.50
0.25
0.00
0
1
2
3
4
5
6
7
8
1m
B
Jmjd6:18m
Frequency
18m
C
Jmjd6:24m
Frequency
24m
D
Jmjd6:30m
Frequency
30m

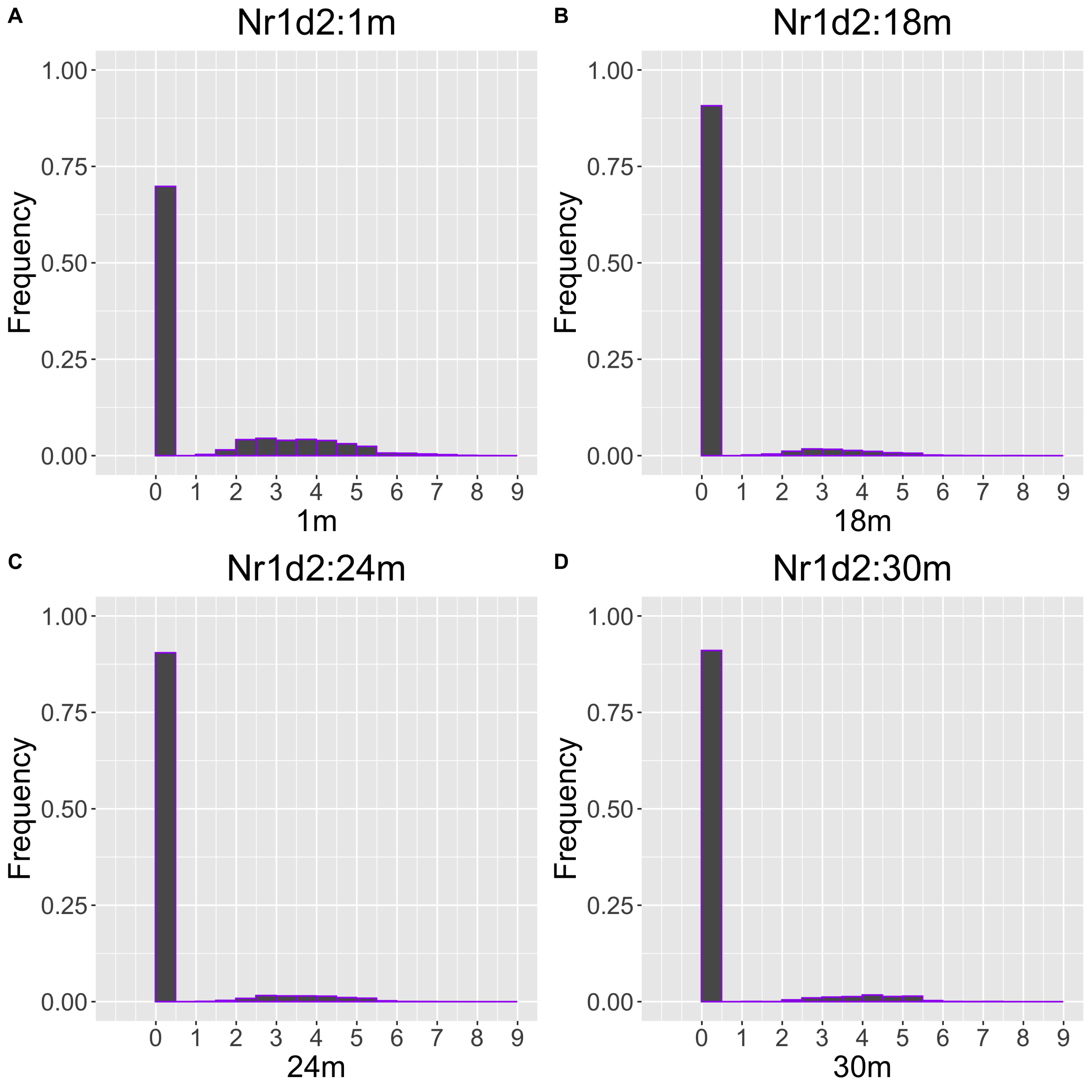
A
Nr1d2:1m
Frequency
1.00
0.75
0.50
0.25
0.00
0 1 2 3 4 5 6 7 8 9
1m
B
Nr1d2:18m
Frequency
18m
C
Nr1d2:24m
Frequency
24m
D
Nr1d2:30m
Frequency
30m

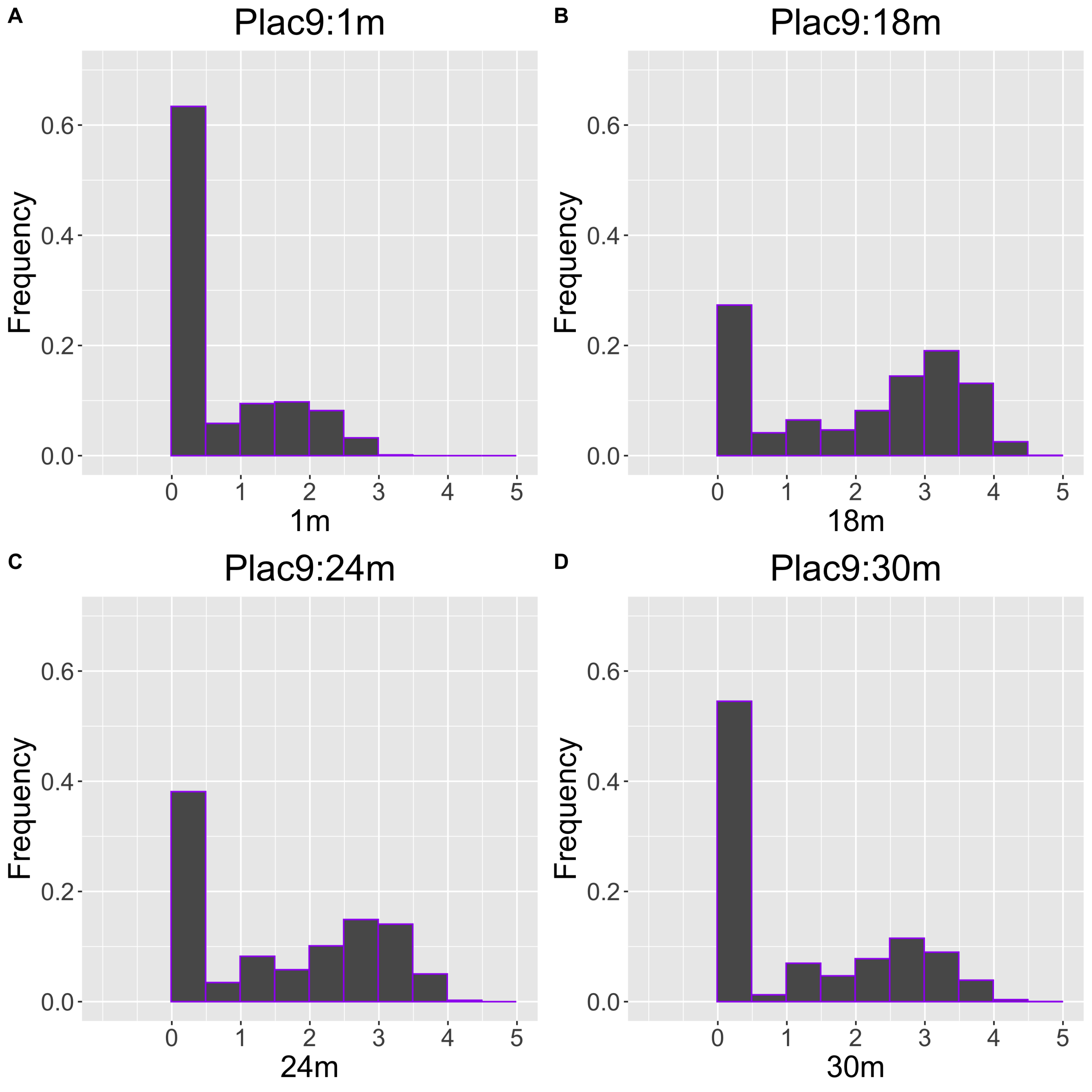
A
Plac9:1m
Frequency
0.0
0.2
0.4
0.6
0
1
2
3
4
5
1m
B
Plac9:18m
Frequency
18m
C
Plac9:24m
Frequency
24m
D
Plac9:30m
Frequency
30m

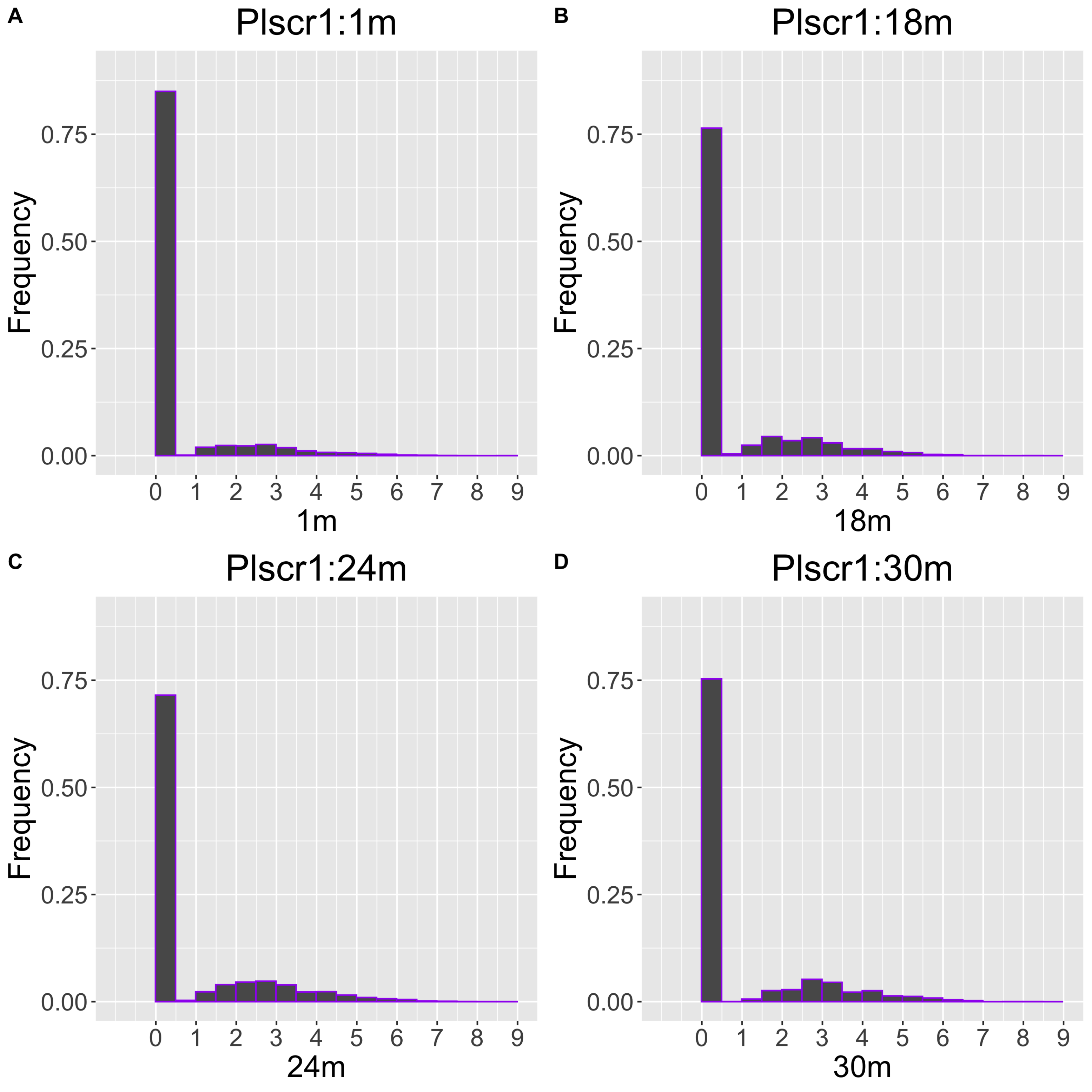

A
Plscr1:1m
Frequency
0.00
0.25
0.50
0.75
0 1 2 3 4 5 6 7 8 9
1m
B
Plscr1:18m
Frequency
0.00
0.25
0.50
0.75
0 1 2 3 4 5 6 7 8 9
18m
C
Plscr1:24m
Frequency
0.00
0.25
0.50
0.75
0 1 2 3 4 5 6 7 8 9
24m
D
Plscr1:30m
Frequency
0.00
0.25
0.50
0.75
0 1 2 3 4 5 6 7 8 9
30m

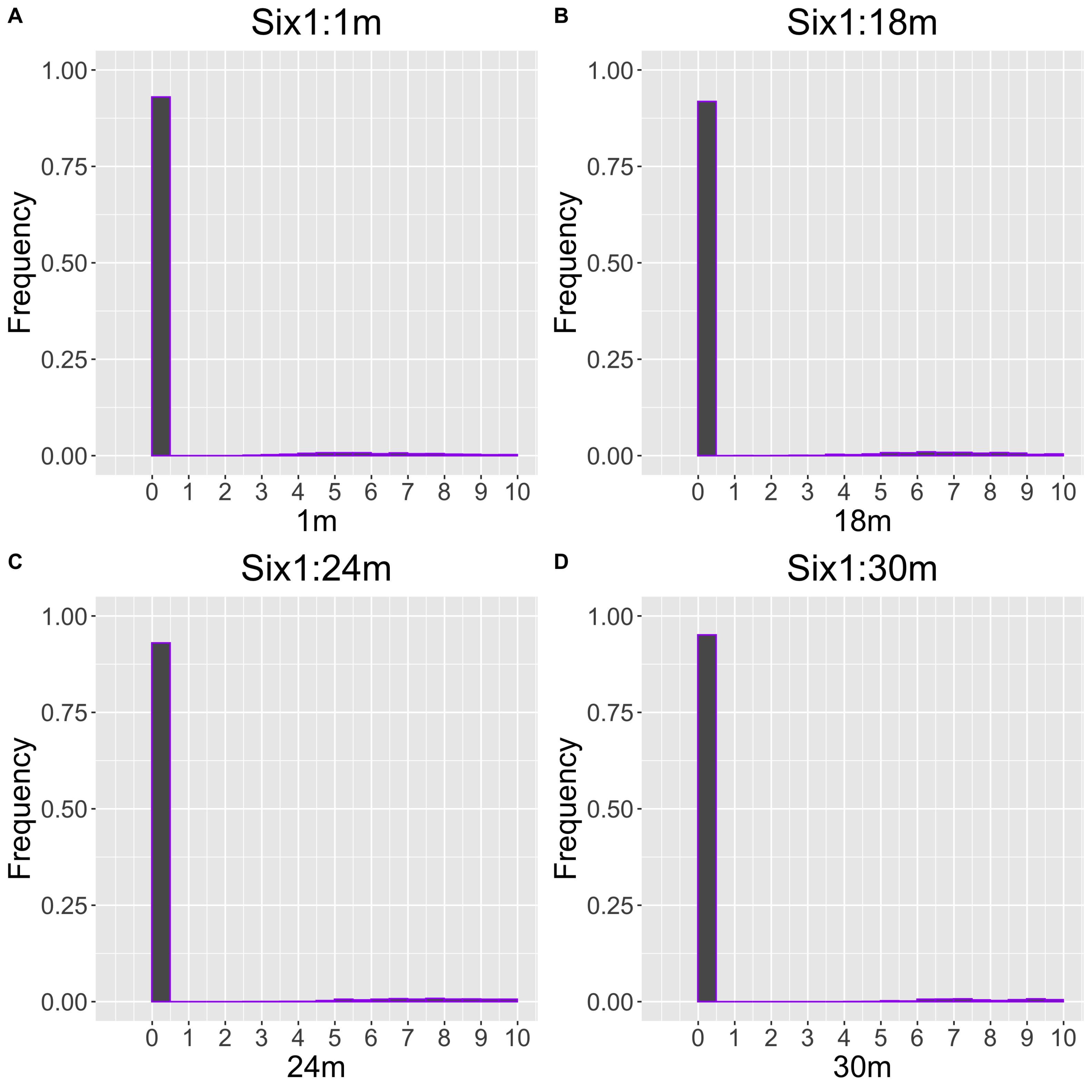

A
Six1:1m
Frequency
1.00
0.75
0.50
0.25
0.00
0 1 2 3 4 5 6 7 8 9 10
1m
B
Six1:18m
Frequency
1.00
0.75
0.50
0.25
0.00
0 1 2 3 4 5 6 7 8 9 10
18m
C
Six1:24m
Frequency
1.00
0.75
0.50
0.25
0.00
0 1 2 3 4 5 6 7 8 9 10
24m
D
Six1:30m
Frequency
1.00
0.75
0.50
0.25
0.00
0 1 2 3 4 5 6 7 8 9 10
30m

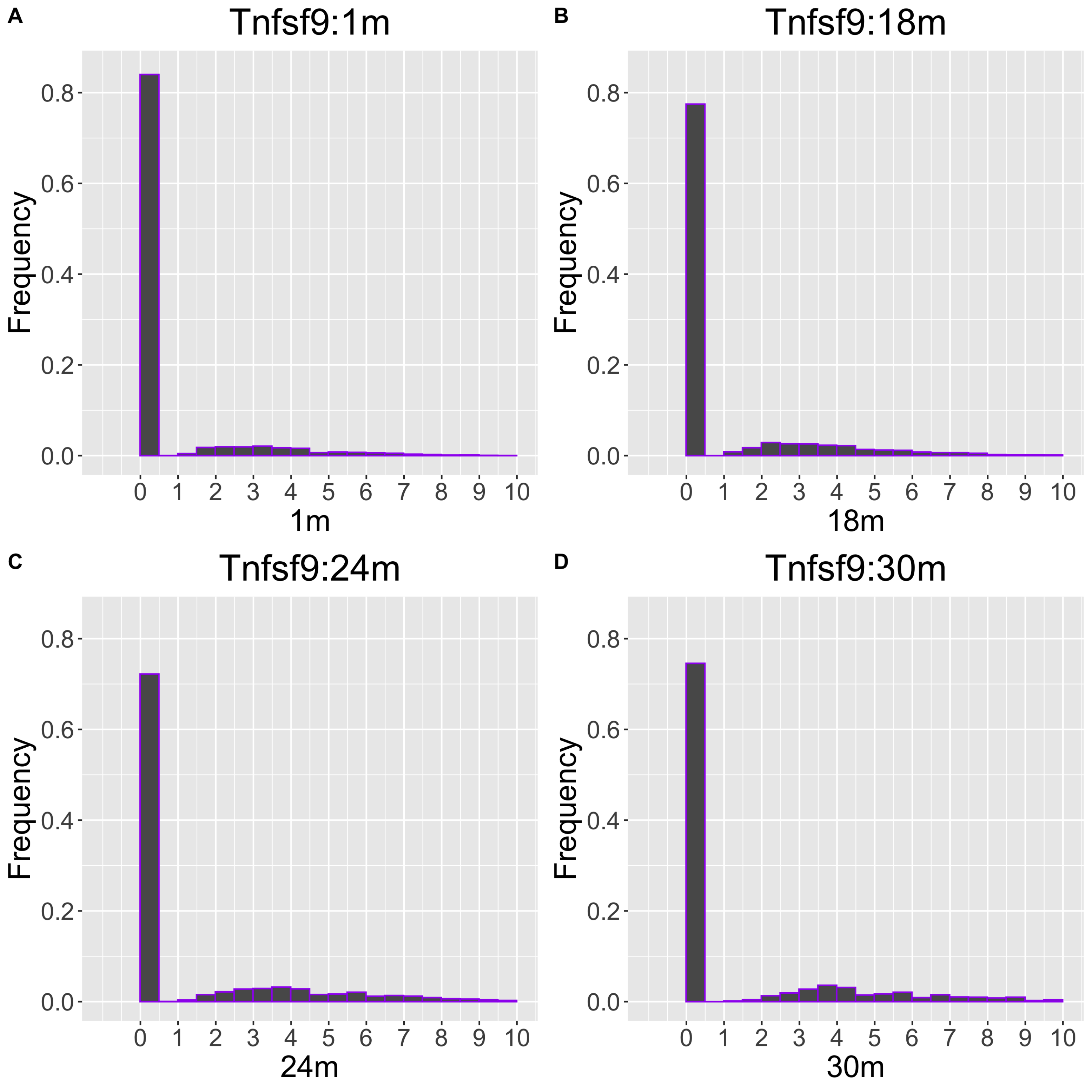

A
Tnfsf9:1m
Frequency
0.0
0.2
0.4
0.6
0.8
0 1 2 3 4 5 6 7 8 9 10
1m
B
Tnfsf9:18m
Frequency
0.0
0.2
0.4
0.6
0.8
0 1 2 3 4 5 6 7 8 9 10
18m
C
Tnfsf9:24m
Frequency
0.0
0.2
0.4
0.6
0.8
0 1 2 3 4 5 6 7 8 9 10
24m
D
Tnfsf9:30m
Frequency
0.0
0.2
0.4
0.6
0.8
0 1 2 3 4 5 6 7 8 9 10
30m

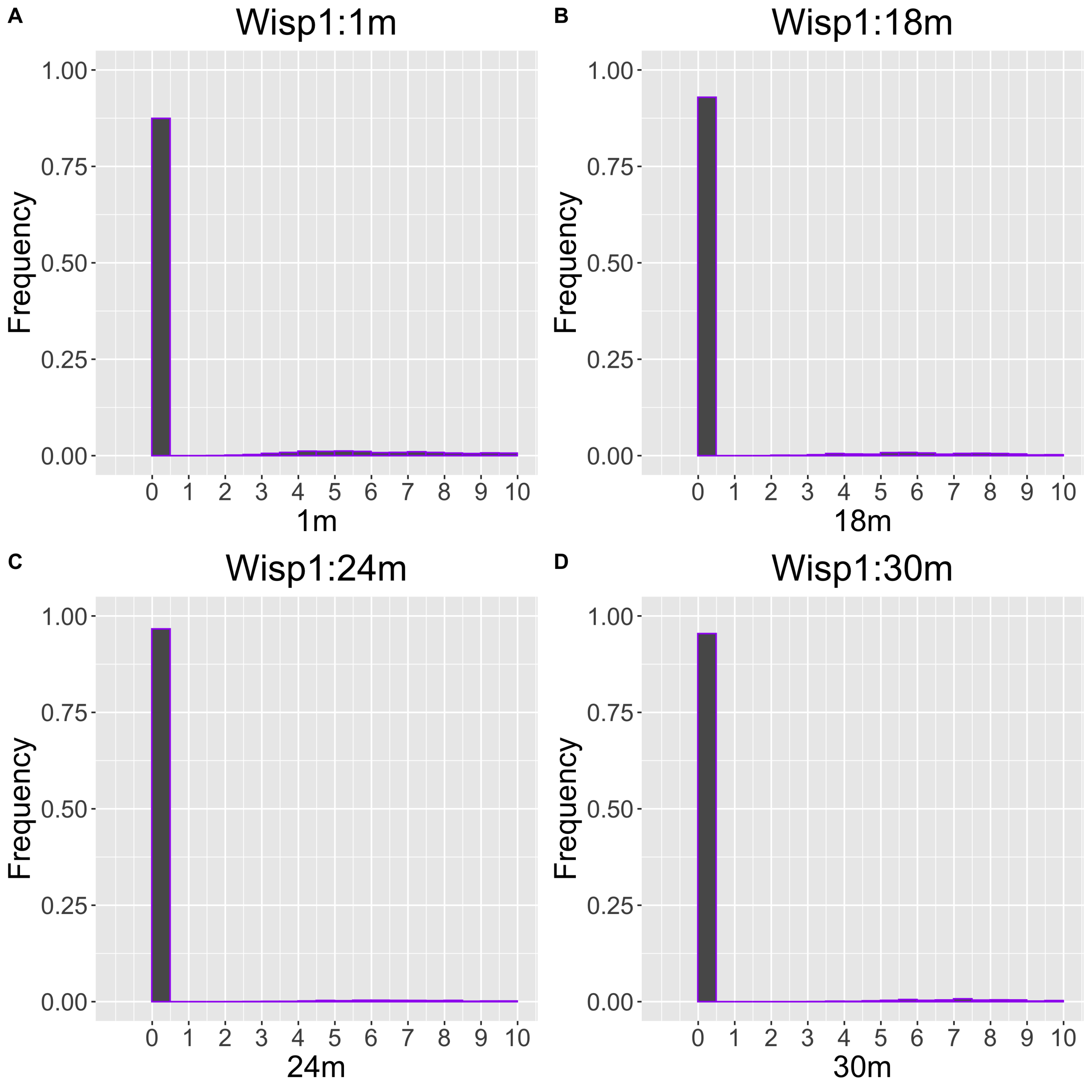
A
Wisp1:1m
Frequency
1.00
0.75
0.50
0.25
0.00
0 1 2 3 4 5 6 7 8 9 10
1m
B
Wisp1:18m
Frequency
1.00
0.75
0.50
0.25
0.00
0 1 2 3 4 5 6 7 8 9 10
18m
C
Wisp1:24m
Frequency
1.00
0.75
0.50
0.25
0.00
0 1 2 3 4 5 6 7 8 9 10
24m
D
Wisp1:30m
Frequency
1.00
0.75
0.50
0.25
0.00
0 1 2 3 4 5 6 7 8 9 10
30m
