# Supplementary material for: Data-driven detection of age-related arbitrary monotonic changes in single-cell gene expression distributions
Source: PeerJ. 2024 Feb 8;12:e16851. doi: 10.7717/peerj.16851 (PMC10859082; doi:10.7717/peerj.16851)
Supplement: Supplemental Information 4 — The x-axis represents the distribution at the youngest age and the y-axis represents the distribution at other ages. Cells from all samples in each age category are pooled. [file peerj-12-16851-s004.pdf]

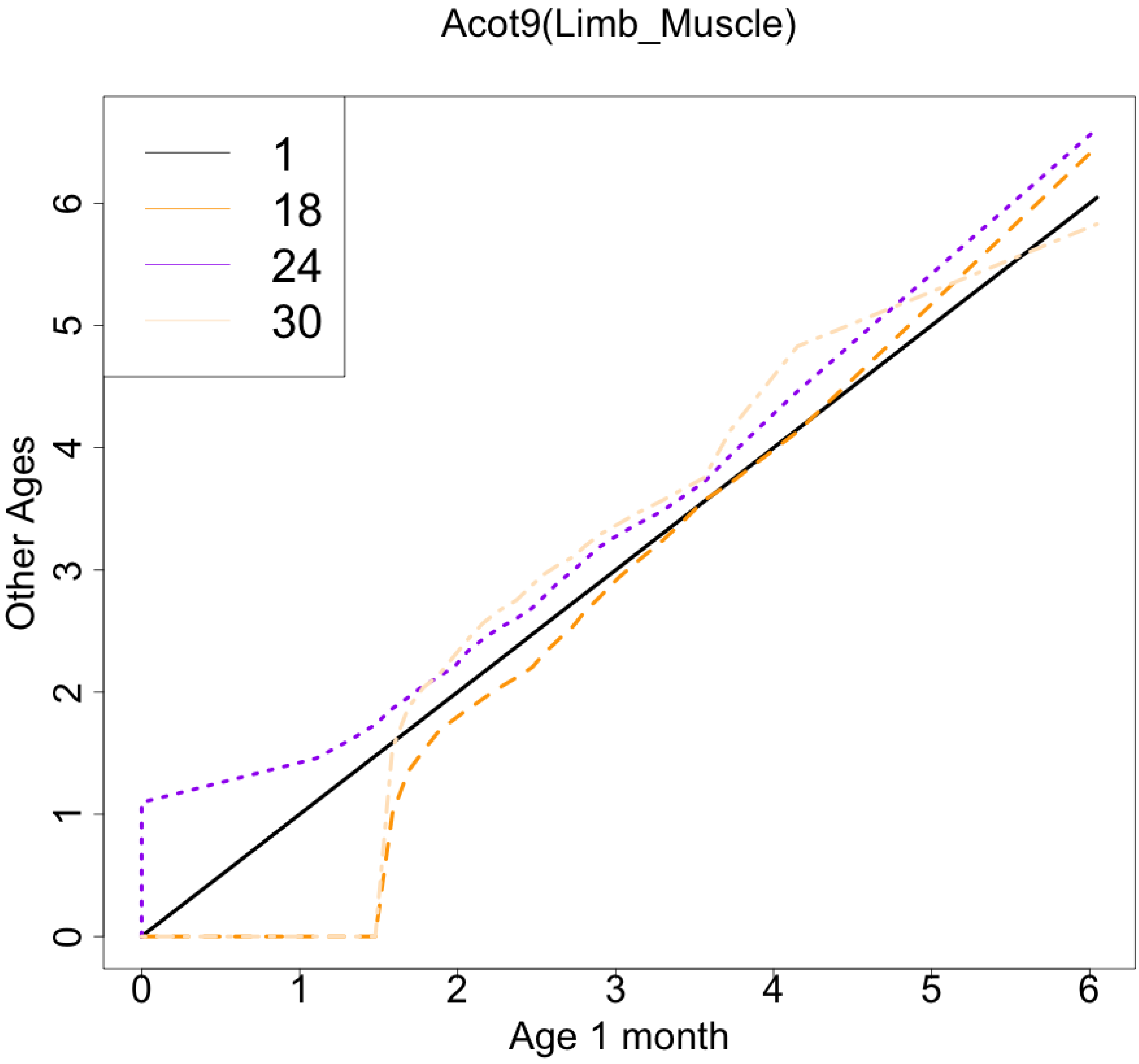

Acot9(Limb_Muscle)
1
18
24
30
0
1
2
3
4
5
6
Other Ages
Age 1 month

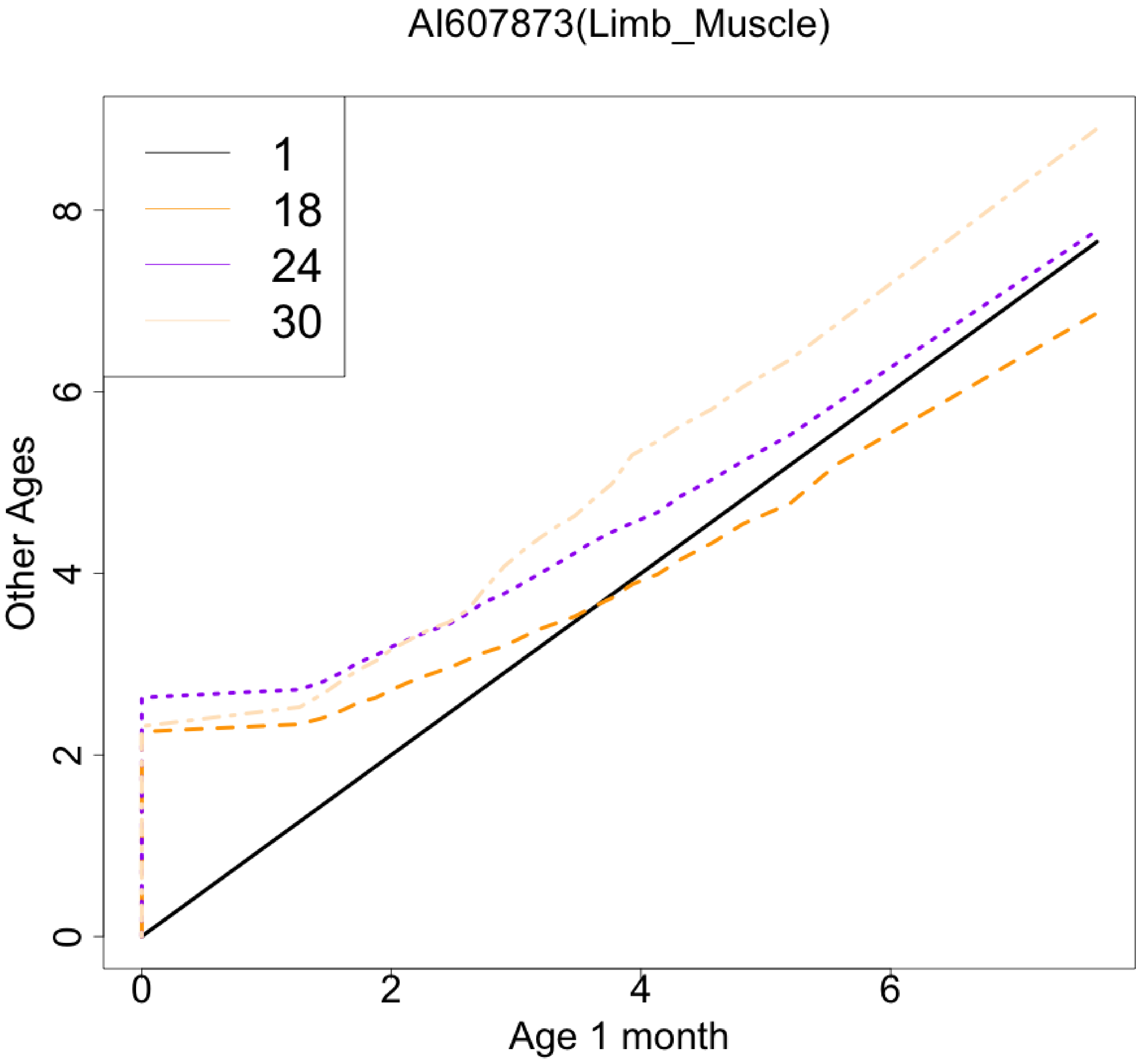
AI607873(Limb_Muscle)
1
18
24
30
Other Ages
Age 1 month
0
2
4
6
8
0
2
4
6

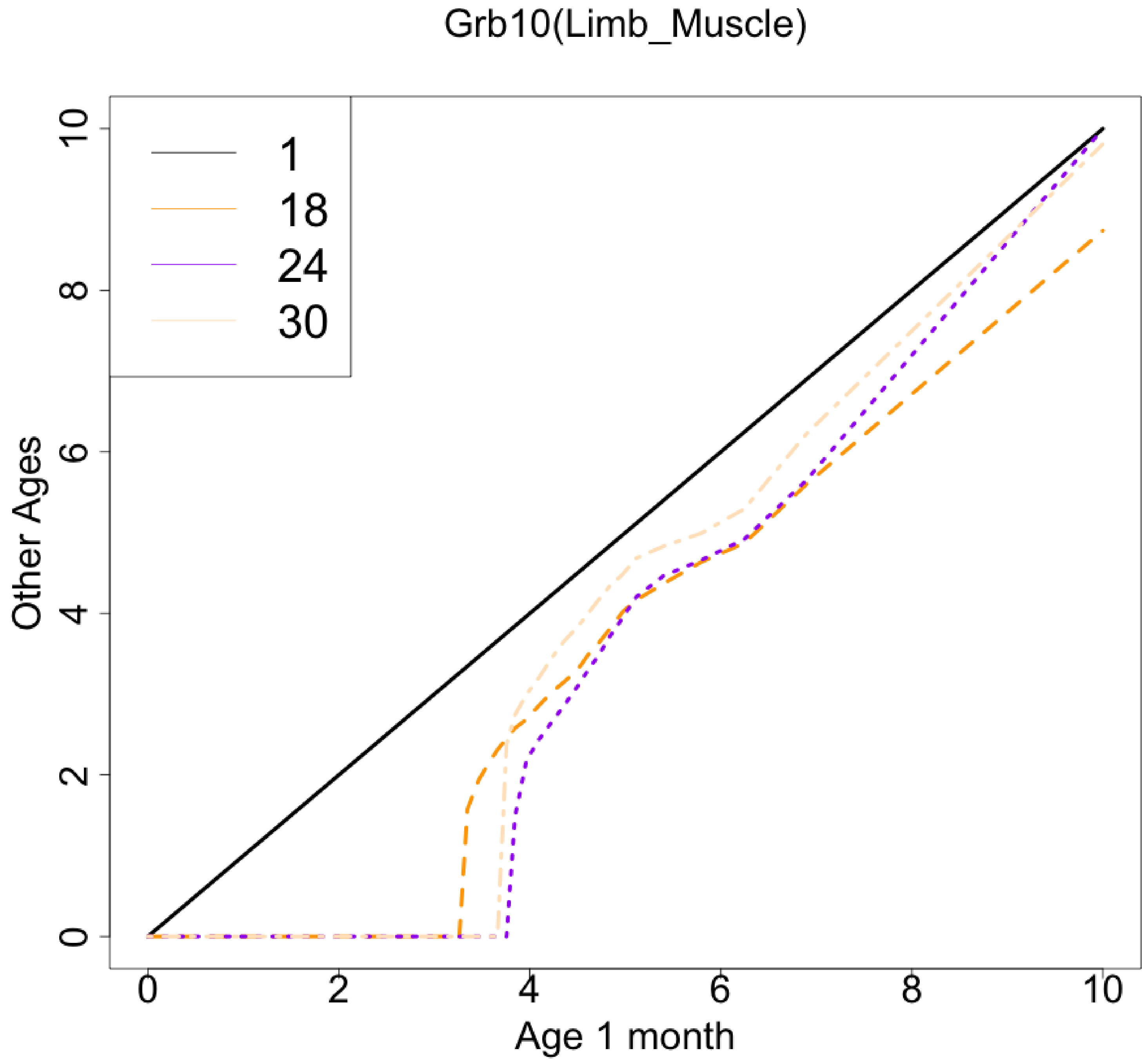

Grb10(Limb_Muscle)
1
18
24
30
Other Ages
Age 1 month
0
2
4
6
8
10

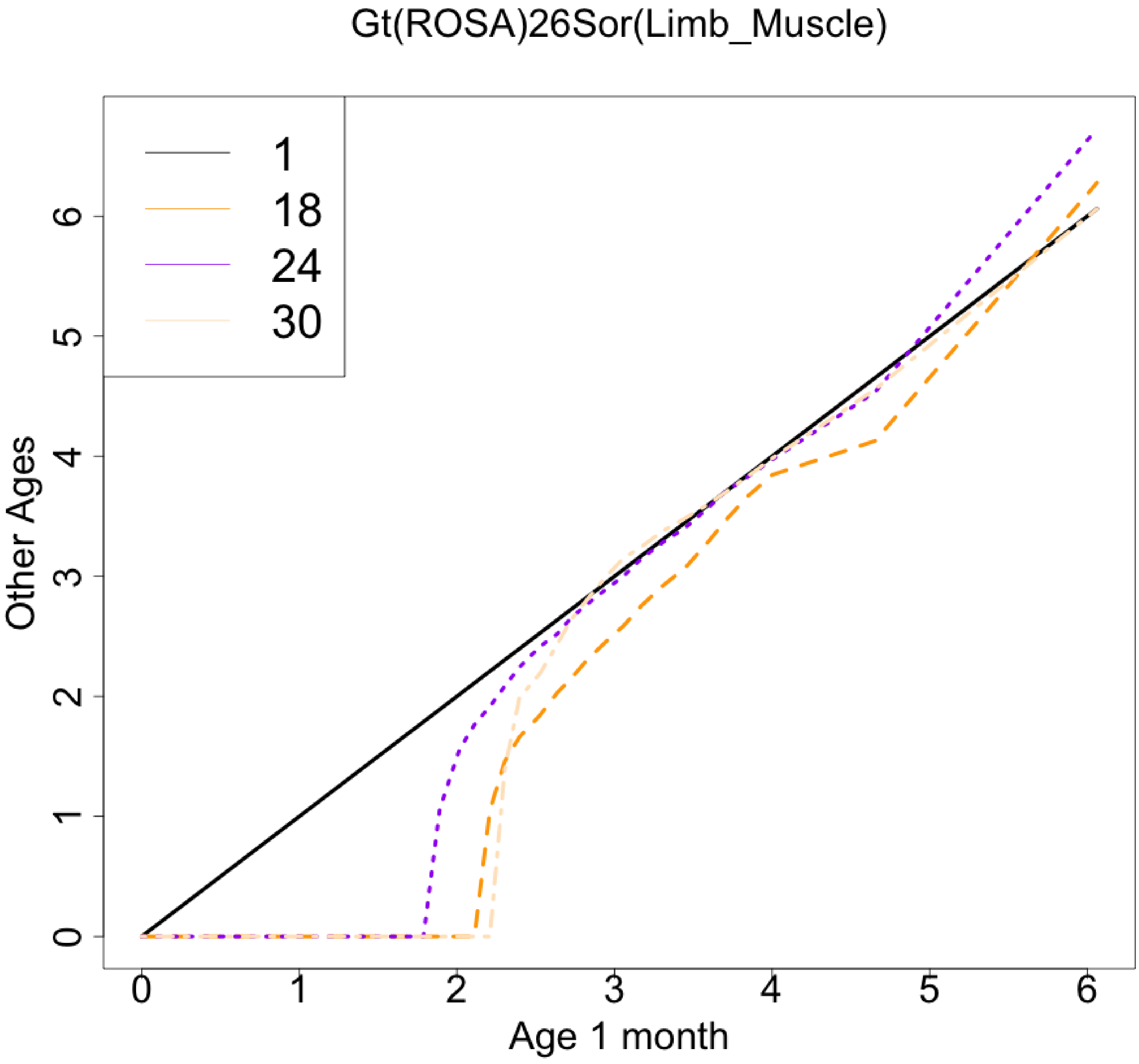

Gt(ROSA)26Sor(Limb_Muscle)
1
18
24
30
Other Ages
Age 1 month
0
1
2
3
4
5
6

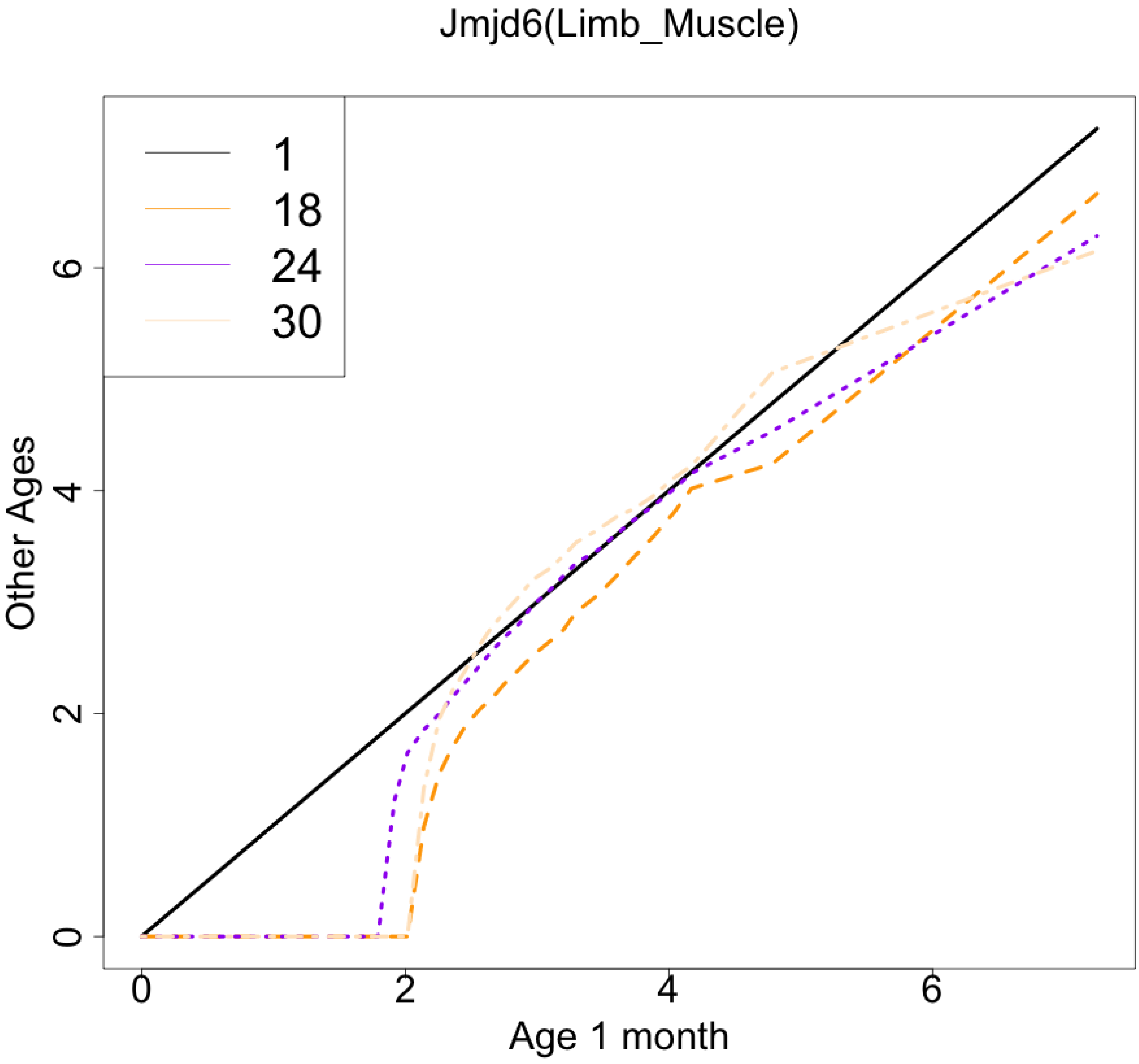

Jmjd6(Limb_Muscle)
1
18
24
30
Other Ages
0
2
4
6
Age 1 month

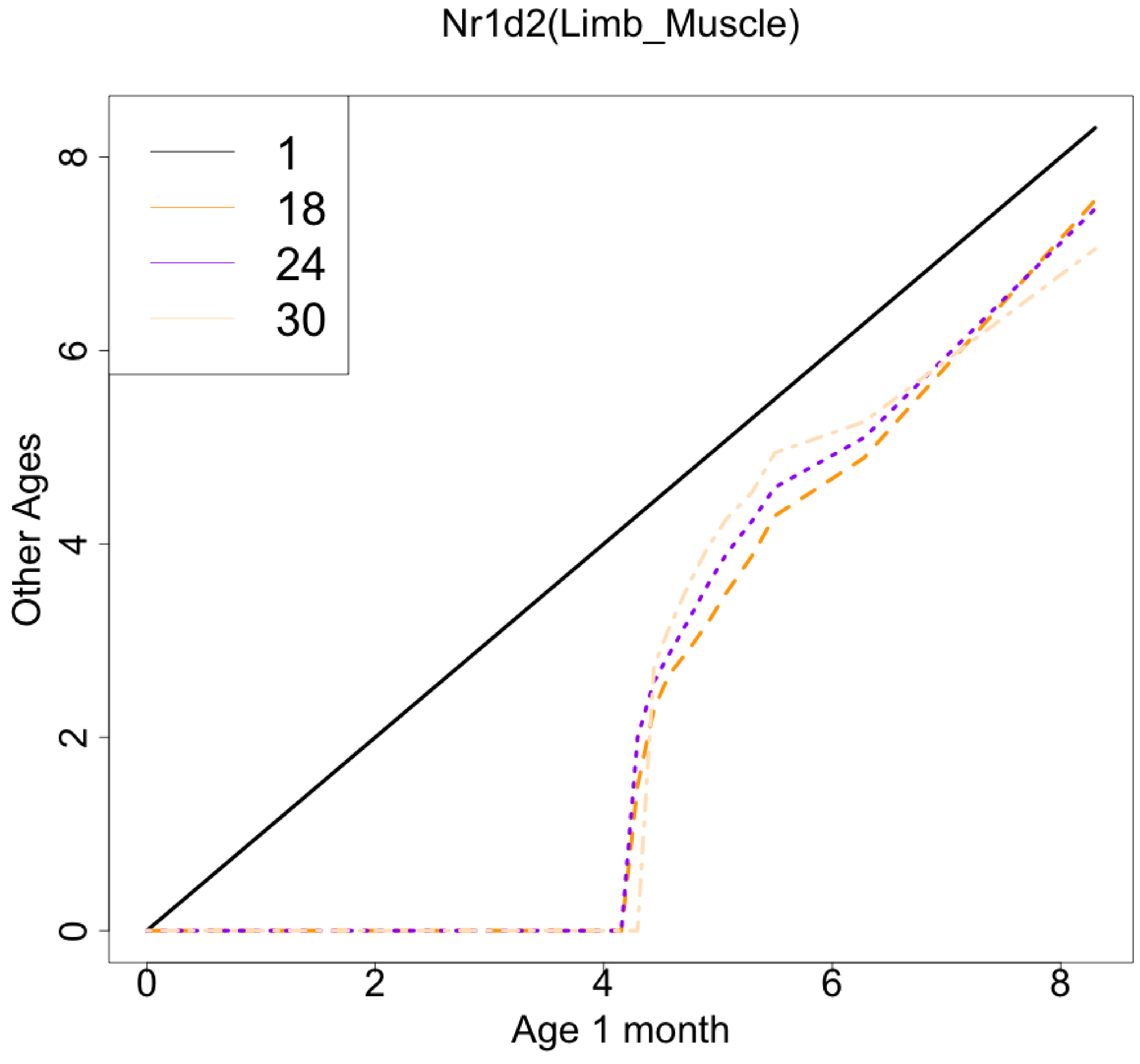
Nr1d2(Limb_Muscle)
1
18
24
30
0
2
4
6
8
Other Ages
0
2
4
6
8
Age 1 month

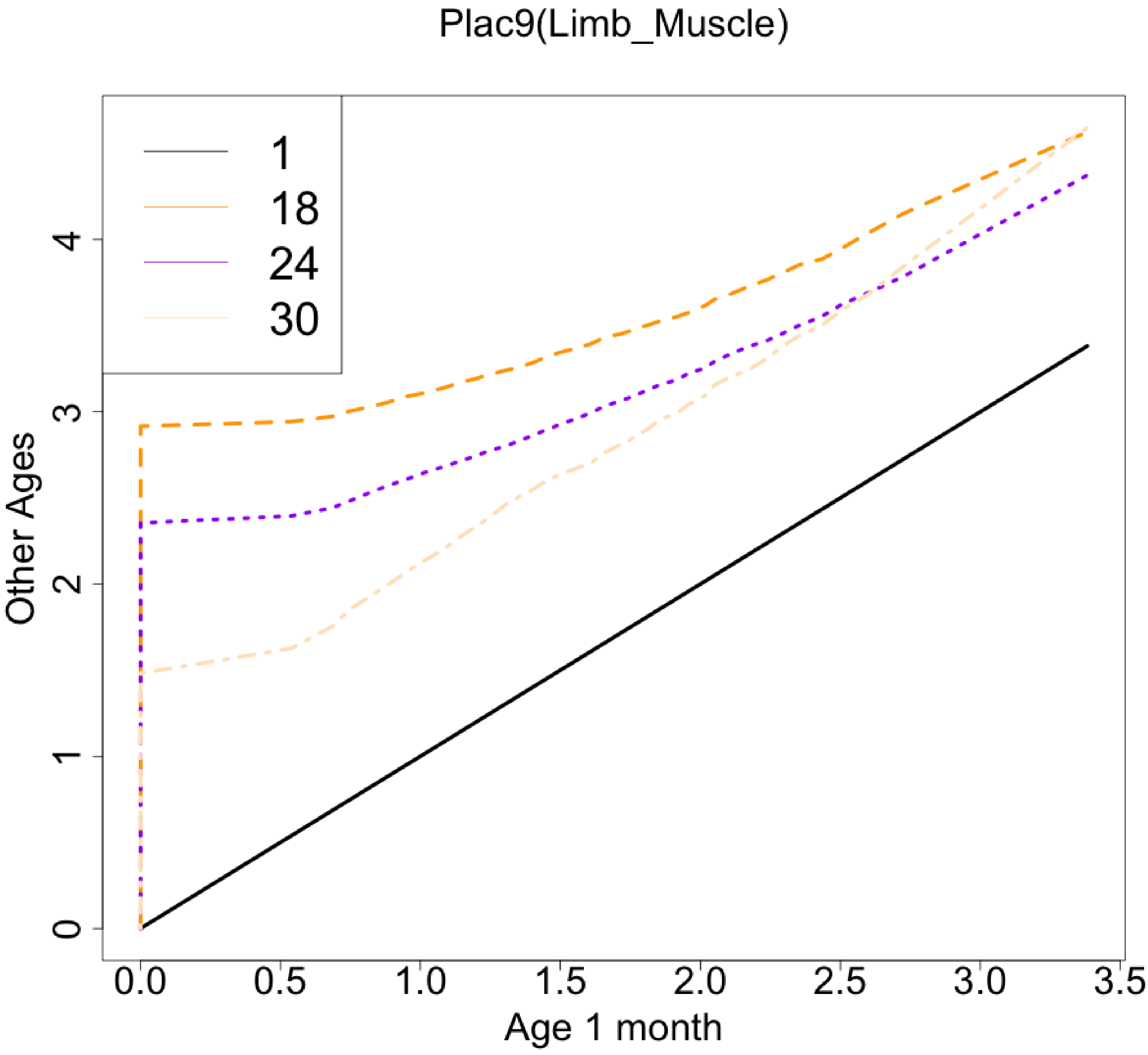
Plac9(Limb_Muscle)
1
18
24
30
Other Ages
0
1
2
3
4
0.0
0.5
1.0
1.5
2.0
2.5
3.0
3.5
Age 1 month

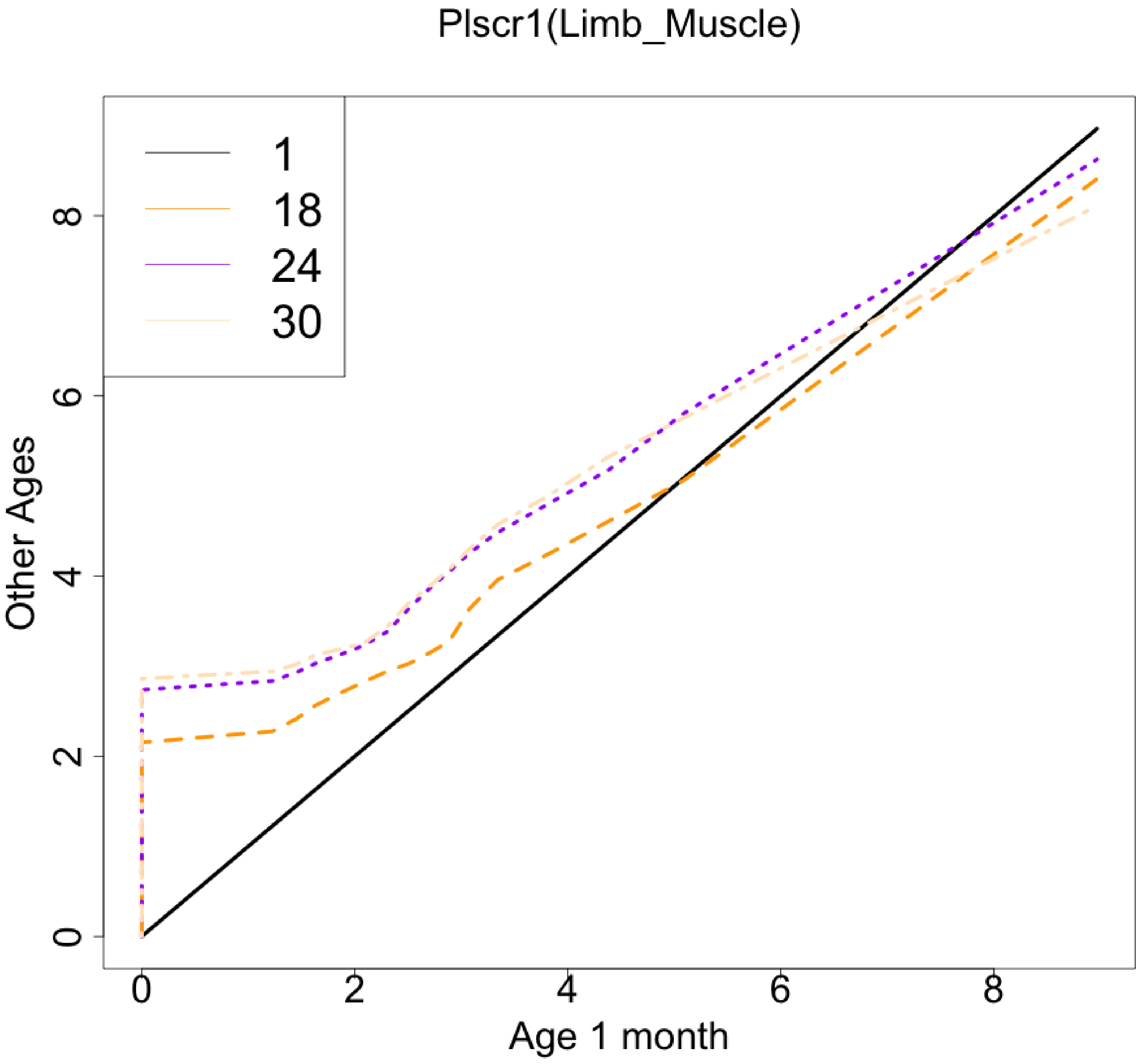

Plscr1(Limb_Muscle)
1
18
24
30
0
2
4
6
8
Other Ages
Age 1 month

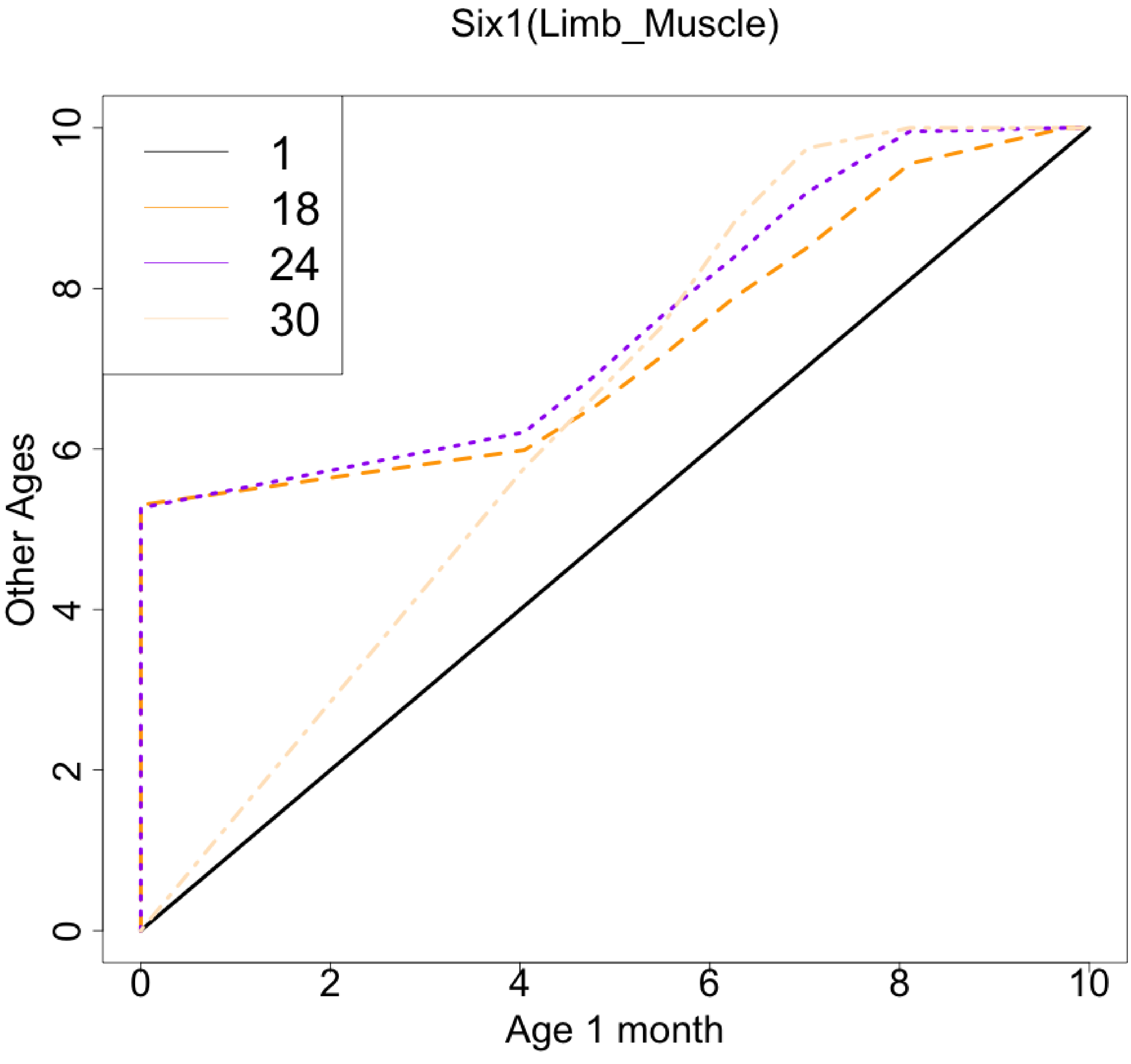

Six1(Limb_Muscle)
1
18
24
30
Other Ages
0
2
4
6
8
10
Age 1 month

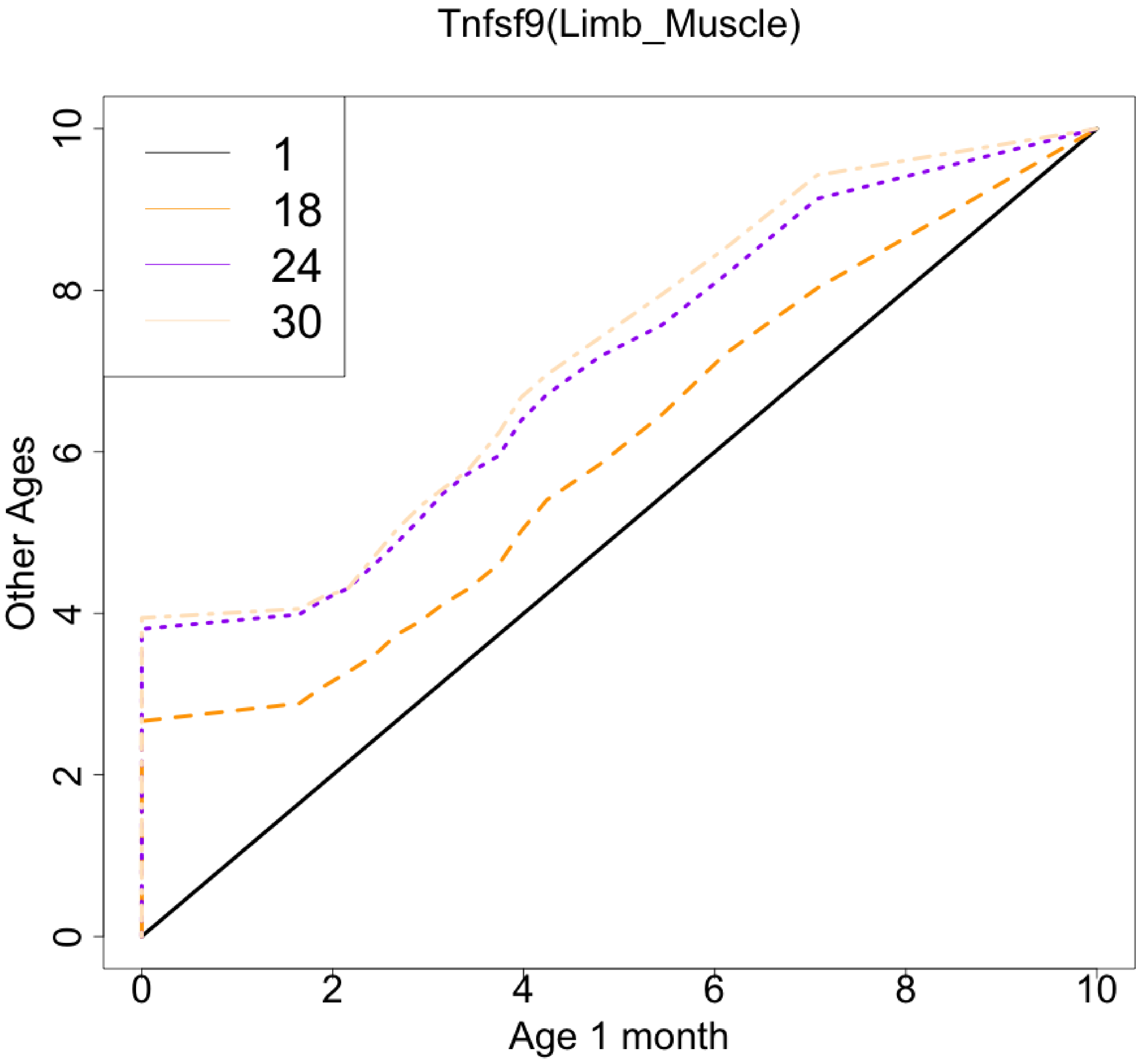

Tnfsf9(Limb_Muscle)
1
18
24
30
Other Ages
Age 1 month
0
2
4
6
8
10
0
2
4
6
8
10

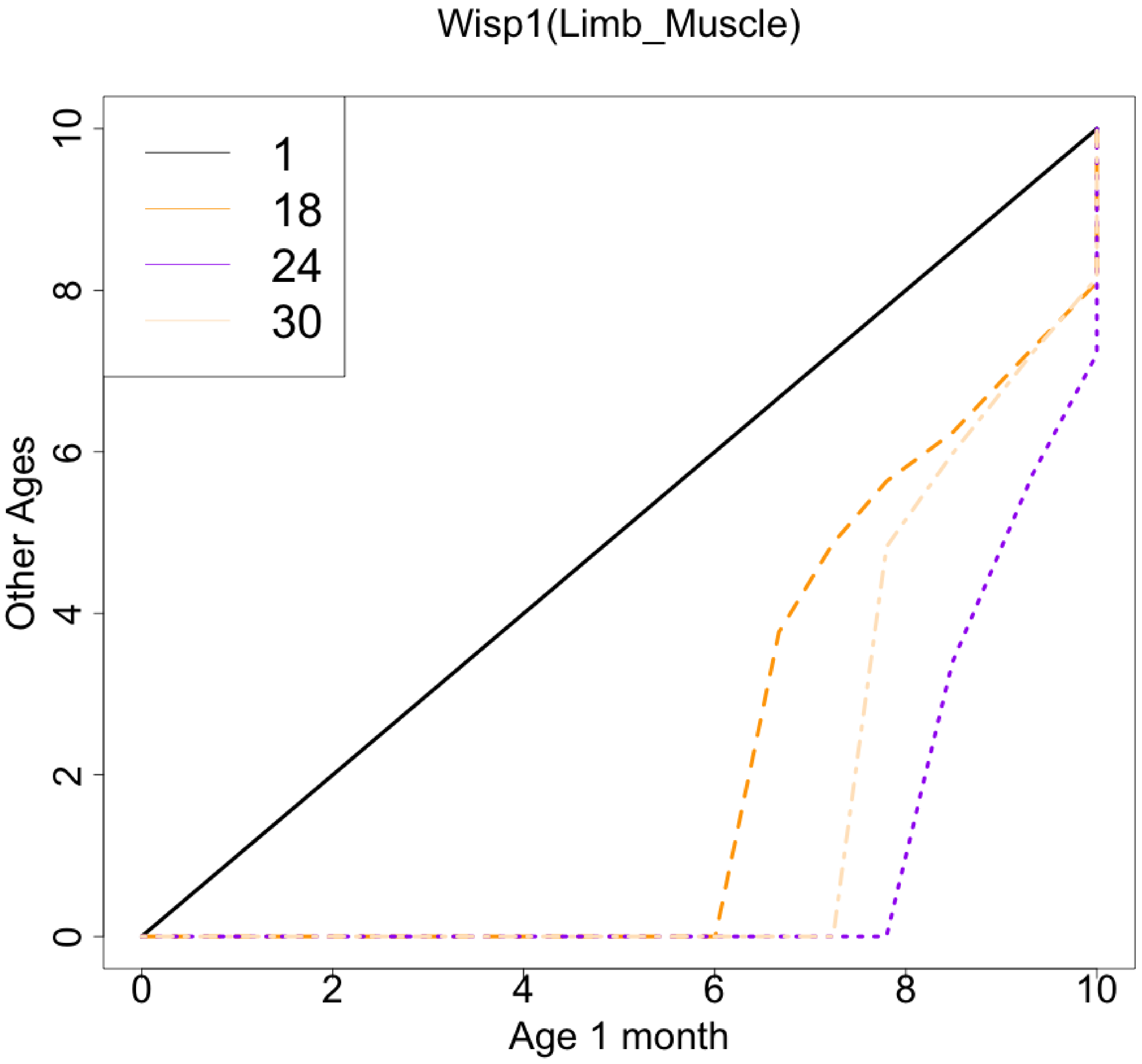

Wisp1(Limb_Muscle)
1
18
24
30
Other Ages
Age 1 month
0
2
4
6
8
10
0
2
4
6
8
10
